# The Intriguing Thyroid Hormones–Lung Cancer Association as Exemplification of the Thyroid Hormones–Cancer Association: Three Decades of Evolving Research

**DOI:** 10.3390/ijms23010436

**Published:** 2021-12-31

**Authors:** Maria V. Deligiorgi, Dimitrios T. Trafalis

**Affiliations:** Department of Pharmacology—Clinical Pharmacology Unit, Faculty of Medicine, National and Kapodistrian University of Athens, Building 16, 1st Floor, 75 Mikras Asias Str, 11527 Athens, Greece; dtrafal@med.uoa.gr

**Keywords:** genomic actions, integrin αvβ3, lung cancer, nongenomic actions, non-small cell lung cancer, non-thyroidal illness syndrome, tetrac, thyroid hormones, thyroid hormone receptors

## Abstract

Exemplifying the long-pursued thyroid hormones (TH)–cancer association, the TH–lung cancer association is a compelling, yet elusive, issue. The present narrative review provides background knowledge on the molecular aspects of TH actions, with focus on the contribution of TH to hallmarks of cancer. Then, it provides a comprehensive overview of data pertinent to the TH–lung cancer association garnered over the last three decades and identifies obstacles that need to be overcome to enable harnessing this association in the clinical setting. TH contribute to all hallmarks of cancer through integration of diverse actions, currently classified according to molecular background. Despite the increasingly recognized implication of TH in lung cancer, three pending queries need to be resolved to empower a tailored approach: (1) How to stratify patients with TH-sensitive lung tumors? (2) How is determined whether TH promote or inhibit lung cancer progression? (3) How to mimic the antitumor and/or abrogate the tumor-promoting TH actions in lung cancer? To address these queries, research should prioritize the elucidation of the crosstalk between TH signaling and oncogenic signaling implicated in lung cancer initiation and progression, and the development of efficient, safe, and feasible strategies leveraging this crosstalk in therapeutics.

## 1. Introduction

With a long history originating with the medical writings of eminent philosophers-physicians of the antiquity, such as Galen (130–200 AD) and Pliny the Elder (23/24–79 AD), the thyroid gland and its products—the thyroid hormones (TH)—still fascinate researchers [1]. ΤH, especially the main active iodothyronines 3,3′,5,5′-tetraiodo-L-thyronine (T4) and 3,5,3′-triiodo-l-thyronine (T3), are indispensable for life through controlling evolutionary conserved pathways. The biological significance of TH is highlighted by identification of precursors of T4 and T3 in a variety of invertebrate species, even in marine algae, despite absence of thyroid tissue [2].

The actions of TH—conventionally classified as genomic and nongenomic—coordinate to integrate several physiological and pathological processes [3]. The physiological processes include fetal and postnatal development, cellular differentiation, oxygen consumption, heat production, free radical formation, and metabolic homeostasis [2]. Among the pathological processes, the neoplastic ones prevail [4].

Originally conceived in 1896 by Beatson, the TH–cancer association remained underexplored for years. The interest for the TH–cancer association was rekindled with the cloning of cDNAs encoding the nuclear TH receptors (TRs) in 1986 [5], which heralded a new realm of investigation. In 1993, the case report of spontaneous remission of metastatic NSCLC following recovery from myxedema coma by Hercbergs and Leith [6] paved the way for the increasing recognition of the implication of TH in a wide array of malignancies beyond the lung, including breast, prostate, ovary, adrenocortical, thyroid, colon, hepatocellular, and pancreatic cancer and melanoma, glioma, glioblastoma, multiple myeloma, lymphoma, sarcoma, and leukemia [4,7,8].

Although the TH-lung cancer association laid the groundwork for pursuing the thyroid gland as intrinsic modifier of cancer [6], it remains elusive, yet important.

Considering the enormous global burden of lung cancer and the challenging management thereof, it would be of interest to clarify the potential exploration of the TH-lung cancer association in the clinical setting.

With 2,206,771 estimated new cases (11.4% of total cases) and 1,796,144 estimated deaths (18.0% of total cases), worldwide in 2020, lung cancer ranks as the second most common cancer and the leading cause of cancer death [9]. Lung cancer patients’ five-year survival rates depend on disease stage at the time of diagnosis, varying from 59.8% for local disease to 32.9% for regional disease, and down to 6.3% for distant disseminated disease [10]. Three histological types of lung cancer have been recognized: (i) non-small cell lung cancer (NSCLC) (80–85%) with 3 subtypes, adenocarcinoma, squamous cell carcinoma, and large cell carcinoma; (ii) small cell lung cancer (SCLC) (15%); (iii) other tumors (e.g., lung carcinoid tumors, lymphomas, sarcomas) [11]. Approximately one third of NSCLC patients are diagnosed with potentially curable early-stage disease [12].

The treatment of lung cancer is based on three pillars: surgical resection, systemic therapy, and radiation therapy. The optimal decision-making is tailored based on disease stage according to the American Joint Committee on Cancer (AJCC) TNM staging system integrated with patient-related and tumor-related characteristics [13,14,15,16,17,18,19,20,21].

Currently, the treatment of lung cancer has been revolutionized by major advances in large-scale molecular profiling of lung tumors unveiling an increasing list of oncogenic drivers exploited as therapeutic targets (e.g., epidermal growth factor receptor [EGFR], vraf murine sarcoma viral oncogene homolog BE [BRAF], rearrangements in anaplastic lymphoma kinase [ALK], proto-oncogene tyrosine-protein kinase ROS1, and neurotrophic receptor tyrosine kinase [NTRK]). A targetable genomic hallmark is identified in up to 69% of patients with advanced NSCLC. Additionally, the regulatory approval of monoclonal antibodies (mAbs) blocking the immune checkpoints, namely, the programmed cell death 1 (PD-1)/PD-1 ligand 1 (PD-L1) pathway and the cytotoxic T-lymphocyte-associated antigen 4 (CTLA-4), known as immune checkpoint inhibitors (ICPi), has altered the survival rates of lung cancer [13,20]. However, the personalization of lung cancer therapeutics remains “a moving target” due to resistance of cancer cells to innovative therapies [21].

The emerging paradigm shift in cancer treatment from targeting single molecular defects to targeting complex molecular interactions [16], leveraging the proteomic landscape of tumors [22], rationalizes capitalizing on the interrelationship of TH signaling with pivotal molecular pathways implicated in lung cancer. Deciphering the TH–lung cancer association is anticipated to refine the management of lung cancer.

The present review suggests three pending queries as the core of a comprehensive overview of the TH-lung cancer association: (1) How to stratify patients with TH-sensitive lung tumors who may benefit from interventions in TH status? (2) How to determine whether TH promote or inhibit lung cancer progression? (3) How to leverage the antitumor effects of TH and/or abrogate the tumor-promoting effects of TH in the clinical setting? These queries set the framework for the present review. The answer to the first two questions entails background knowledge about the synthesis, metabolism, and transport of TH addressed in Section 2. Additionally, it should be sought in the molecular background of the crosstalk between TH signaling and oncogenic signaling in general—addressed in Section 3 and Section 4—and in lung cancer—addressed in Section 5.1—with the hope of identifying and validating predictive biomarkers and risk factors as discussed in Section 7. The answer to the third question should be sought in the understanding of the clinical aspects of the TH-lung cancer association—addressed in Section 5.2 and Section 6—and the potential development of efficient, safe, and feasible strategies lever-aging this crosstalk in therapeutics—addressed in Section 7.

## 2. Synthesis, Metabolism, and Transport of TH

T4 is the prevailing iodothyronine and the only one derived exclusively from secretion from thyroid gland. Thyroid gland produces and releases also T3, but almost 80% of circulating T3 arises from the removal of a single 5 iodine atom from T4 (outer ring or 5′ monodeiodination) in tissues outside thyroid gland [2,23].

The biosynthesis of TH is a multistep process requiring: (i) active iodine transport via sodium/iodide symporters (NIS) into follicular thyrocytes, (ii) iodide oxidation, (iii) covalent linkage of iodine to tyrosine residues of thyroglobulin to produce first MIT and then DIT, (iv) coupling of two molecules of DIT to form T4, and (v) coupling of one molecule of MIT with one molecule of DIT to form T3. The iodination of thyroglobulin and the coupling of iodotyrosyl residues in Tg are catalyzed by thyroid peroxidase (TPO) [2,24].

Three deiodinase (DIO) enzymes (DIO1, DIO2, DIO3) with differing tissue localization, substrate specificity, and physiologic and physiopathologic regulation are credited with maintenance of TH homeostasis, as depicted in Table 1 [2,24].

Currently, this historical landscape is being enriched with several derivative metabolites of T4 and T3, generated through different classes of enzymes, beyond DIO, namely amine transferases, amine oxidases, decarboxylase, and sulfotransferases. Such metabolites are designated as novel TH and include the 3,3′,5′-triiodothyronine or reverse T3 (rT3), the 3,5-diiodothyronine (T2), the thyronamines (e.g., the 3-iodothyronamine [T1AM] and the non-iodinated thyronamines [T0AM]), the thyroacetic acids (e.g., the 3,5,3′,5′-thyroacetic acid [TA4], the 3,5,3′-thyroacetic acid [TA3], and the 3-thyroacetic acid [TA1]) [25]. Similarities and differences between TH and their metabolites have been increasingly identified [26]. Once considered inactive, the TH derivatives are nowadays acknowledged as biological active compounds with potential clinical applications [27,28,29,30,31,32].

In the blood, T4 and T3 are almost entirely bound to plasma proteins, i.e., thyroidbinding globulin (TBG), transthyretin (TTR), and albumin. Only the unbound (free [f]) forms of TH are available to tissues. Once released by thyroid gland, TH entry into cells either by diffusion or by specific carrier mechanisms mediated by membrane transporter proteins, such as monocarboxylate transporter (MCT) 8, MCT 10, organic anion transporter protein-1c1 (OATP1c1), and nonspecific L-type amino acid transporters 1 and 2 (LAT1, LAT2). The expression profile of TH transporters is tissue-specific [33,34].

Historically, the hypothalamic-pituitary-thyroid (HPT) axis has been considered as the tuner of the circulating levels of TH through negative feedback mechanisms. Briefly, hypothalamus secretes thyroid releasing hormone (TRH), which stimulates pituitary to secrete thyroid stimulating hormone (TSH), which, in turn, stimulates TH synthesis and secretion from thyroid. TH, especially T3, inhibit TRH and TSH secretion [2]. However, very recently, the HPT axis has been suggested as a dynamic system with plasticity, allowing adaptation to demanding situations through regulating “life-history trade-offs between reproduction, growth, immunity and basal metabolic rate” in the setting of an evolutionary ecology framework [35].

## 3. Molecular Aspects of the Actions of TH

The actions of TH are an integration of conventionally considered genomic (classical) and nongenomic (nonclassical) actions. The conventionally considered genomic actions of TH are exerted through direct regulation of transcription, mediated by nuclear TRs. Given that the genomic actions require synthesis of RNA and proteins, a time lag between effect and result is justified. More than 40 years ago, innovative studies revealed rapid nonclassical actions of TH implicated in calcium efflux [36,37], glucose uptake [38], actin polymerization [39], and mitochondrial function [40]. Such actions were initially considered transcription-independent, and thus, they were nominated as nongenomic.

Currently, major advances in our understanding of TH actions [2] indicate the need for revolutionizing the relevant nomenclature. Indeed, many of the conventionally considered nongenomic effects can eventually affect transcription and may even be mediated by TRs located at the plasma membrane or in the cytoplasm, as well as by alternatively spliced TRs isoforms [32]. Additionally, the direct action of T3 on mitochondrial function cannot be considered nongenomic because it affects the genome of mitochondria. Finally, the tenet that a rapid TH action cannot implicate genomic signaling is incorrect because rapid post-translational changes, such as the phosphorylation of TRs, can rapidly regulate gene expression mediated by chromatin-bound TRs. In that respect, a novel classification of TH effects in four types, based on involved molecular pathways, has been proposed [41].

Type 1 comprises the TRs-dependent TH actions, induced by recruitment of TRs to chromatin [5,41]. TRs are members of the nuclear receptors’ superfamily, which consists of ligand-activated transcription factors that control development, differentiation, and metabolism in a tissue-specific and cell-specific manner [42]. Two distinct genes—the cellular c-erbA α and c-erbA β genes on human chromosomes 17 and 3, respectively—encode two distinct types of TRs (TRalpha [TRα] and TRbeta [TRβ], respectively). Alternative splicing generates various TRs isoforms with similarities and differences. The T3-binding TRα isoforms are the TRα1, the three TRβ (β1, β2, and β3), as well as the truncated TRΔα1, TRΔα2, and TRΔβ3 [43]. The tissue distribution of TRs isoforms varies considerably: TRα isoforms prevail in heart, brain, and bone, while TRβ isoforms prevail in liver, kidneys, pituitary gland, and brain.

TRs interact with thyroid response element(s) (TRE(s)), which are specific DNA sequences in the regulatory regions of the TH target genes, formed by two half-sites, each including at least the AGGTCA hexamer consensus motif, separated on average by four nucleotides. Although TRs can bind to chromatin as monomers or homodimers, they often bind to TREs as heterodimers with the retinoic acid X receptor (RXR). Distinct species (TR, TR/TR, and RXR/TR) show preference for different TREs.

Unliganded TRs bind to TREs and form co-repressor complexes including the nuclear corepressor (NCoR), the silencing mediator for RXR and TR (SMRT), and certain histone deacetylases (HDAC). The latter are also associated with methyl-CpG-binding proteins, thereby contributing to methylation-dependent gene silencing. Binding of TH to TRs leads to alteration of the conformation of TRs, resulting in dissociation of corepressors (NCoR or SMRT), recruitment of transcriptional coactivators, and induction of target gene transcription. Especially, the TH-bound TRs form co-activator complexes that include the nuclear coactivator 1 (NCoA-1), the transcriptional intermediary factor 2 (TIF2/GRIP-1/NCoA-2), the cAMP-response element binding protein (CREB)-binding protein (CBP), also known as p300, the p300/CBP-associated factor (p/CAF), the vitamin D receptor-interacting protein/TR-associated protein (DRIP/TRAP), and the mediator complex subunit 21 (MED/21, also known as Srb7). Rarely, the TH-bound TRs can repress transcription through unknown mechanisms, likely due to inhibition of other transcriptional factors. Several studies showed that the T3-TRs-TREs signaling can also affect the expression of miRNAs [44].

A critical step for the regulation of TRs action is their trafficking between nucleus and cytoplasm. Interaction of the nuclear transport proteins importin 7, importin β1, and the adapter importin α1 with the nuclear localization motifs (NLS1 and NLS2) of the receptor is responsible for the nuclear entry of TRα1. TRβ1, which lacks NLS-2 and is located initially in the cytoplasm, is translocated to nucleus by the importin α1/β1 heterodimer [45].

Type 2 comprises the TRs-dependent actions of TH mediated by indirect binding of TH-bound TRs to DNA. Experiments based on chromatin immunoprecipitation and sequencing (ChIP-Seq) demonstrated that TRs can interact with chromatin indirectly by tethering to other chromatin proteins. Although there is a paucity of relevant biochemical evidence, examples of type 2 TH actions, especially of T3, are the modulation of the activator protein (AP) 1 (AP1) (jun/fos) and the inhibition of expression of genes encoding the β subunit of TSH (TSHβ) [46,47,48,49] and the DIO2 [50] in the pituitary.

Type 3 comprises the TH actions that are independent of direct or indirect binding to DNA. Such actions are mediated through interaction of cytoplasmic TRs with kinases normally cited at the plasma membrane, resulting in activation of signaling pathways, such as the phosphatidylinositol 3-kinase (PI3K) pathway, which in turn activates the mammalian target of rapamycin (mTOR), promoting transcription. At the plasma membrane, T3 interacts with the truncated TRα isoforms p30 TRα1—a transcriptionally incompetent protein translated from an internal AUG codon of the TRα1 messenger RNA [3]—to stimulate the binding of PI3K to p85α, resulting in inactivation of transcription of down-stream genes. On the other hand, the T3-liganded TRα1 activates a series of signal trans-duction proteins (e.g., type II cGMP-dependent protein kinase [PKGII] and extracellular signal-regulated kinase [ERK]) and the endothelial nitric oxide synthase (NOS) [8]. In the cytoplasm, T3 interacts with TRβ1 to activate rapidly the PI3K pathway and the down-stream transcription of target genes, including hypoxia inducible factor-1α (HIF-1α), glucose transporter 1 (GLUT1), platelet-type phosphofructokinase (PFKP) and monocarboxylate transporter 4 (MCT 4) [8].

Type 4 comprises the nuclear TRs-independent TH actions. A typical example of this type is the binding of T4 and T3 to the membrane TR at integrin αvβ3, as described for the first time in 2004 by Davis et al. [51,52].

Integrin αvβ3 is a heterodimeric plasma membrane protein credited with cell-cell and cell-extracellular matrix (ECM) protein interactions, which contains an Arg-Gly-Asp (RGD) recognition specific binding site for ECM proteins, such as osteopontin, fibronectin and vitronectin. The downstream signaling is mediated through various kinases and is implicated in cytoskeletal organization [53,54], cell motility [55,56,57,58,59], endocytosis [56], and gene transcription [57,58,59].

Integrin αvβ3 is amply expressed in cancer cells and in rapidly dividing endothelial cells. Beyond interacting with ECM proteins, integrin αvβ3 contains specific receptors for TH [52,60], dihydrotestosterone, and resveratrol [61]. The signal transduction down-stream of the binding of TH to the TR near the RGD site of integrin αvβ3 regulates the division of cancer and endothelial cells and determines the fate of cancer cells [62].

Integrin αvβ3 contains two TH binding sites, S1 and S2, which activate different downstream pathways. S1 binds T3 and activates the PI3K/Akt/protein kinase B (PKB) pathway via Src kinase activation. Downstream consequences are the shuttling of cytoplasmic TRα to the nucleus and the increase of expression of target genes, such as HIF-1α. S2 binds T4 and, with lower affinity, T3 and activates PI3K/Akt and mitogen-activated protein kinase (MAPK)/ERK1/2 pathways via phospholipase C (PLC) and protein kinase Cα (PKCα) [8]. At physiological concentrations, T4 is the principal ligand of S2 [63].

Downstream of the binding of T3 or T4 to S2 at integrin αvβ3, the MAPK/ERK1/2 and PI3K/Akt signaling cascades are activated to induce: (i) upregulation of genes expressing several proteins, such as proliferating cell nuclear antigen (PCNA), HIF-1α, p21, activinβC, thrombomodulin, sineoculis homeobox homolog 1 (SIX1), RAS Guanyl Releasing Protein 3 (Rasgrp3), N-myc downstream-regulated gene 2 (Ndrg2), lipocalin 2, B-lymphoma Moloney murine leukemia virus insertion region-1 (BM1) [8], tryptophan biosynthesis protein (TRP1), ERM, Signal transducer and activator of transcription 1(STAT1) and p35 [63]; (ii) activation of mesenchymal-epithelial transition (MET)/focal adhesion kinase (FAK) and nuclear factor-κB (NF-κΒ) signaling cascades [8]; (iii) downregulation of cyclin-dependent kinase 2 (CDK2), cyclin E, follistatin, and phosphorylation of retinoblastoma (Rb) protein. Overall, tumor-promoting signals are transduced [8]. Additionally, TH-activated MAPK induces the sodium proton exchanger (Na+/H+) [8] and increases the activity of the sodium pump (Na, K-ATPase) [62]. Moreover, T4-activated ERK 1/2 modulates intracellular protein trafficking of estrogen receptor alpha (ERα) and TRβ1 from the cytoplasm to nucleus [8].

The integrin αvβ3-mediated TH actions that are relevant to cancer biology can be regulated by the 3,3′,5,5′-tetraiodothyroacetic acid—a deaminated form of T4 known as tetrac—and its nanoparticulate analog—known as nano-diamino-tetrac (NDAT) or tetrac-NP [62,63,64,65,66,67,68,69,70,71]. Tetrac is a monocarboxylic acid carrying four iodo substituents at positions 3, 3′, 5 and 5′. Tetrac binds to nuclear TRs with low affinity, thereby acting as a low-grade thyromimetic in the nucleus. However, tetrac blocks the TH signaling initiated at integrin αvβ3, acting as antagonist of TH actions at this cite [66]. Additionally, tetrac and NDAT act at integrin αvβ3 to mediate trafficking of proteins from cytoplasm to nucleus, nucleoprotein phosphorylation, and generation of nuclear coactivator complexes pertinent to genomic actions of T3 [66].

**Figure 1:**
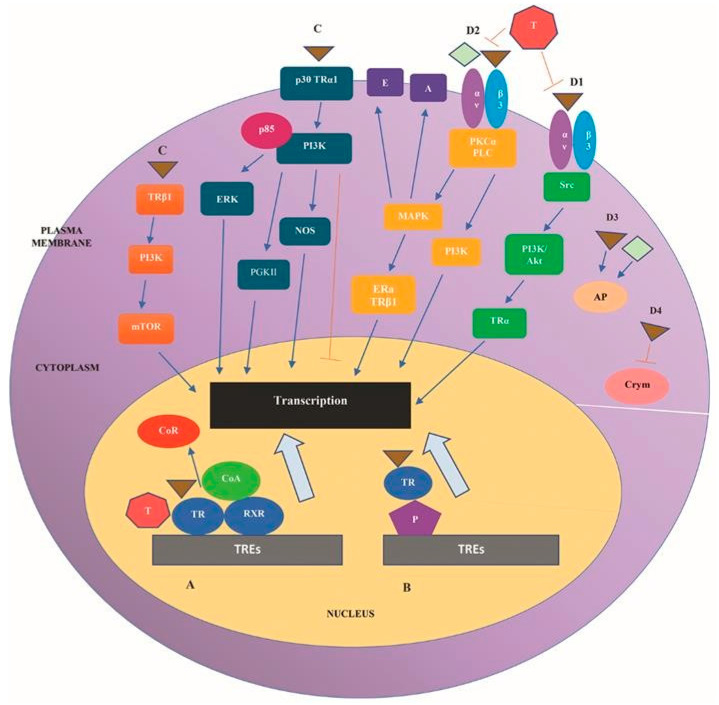
The main signaling cascades implicated in the four types of TH actions. A. Type 1 comprises the nuclear TRs-dependent TH actions, induced by recruitment of TRs to TREs. Binding of T3 (represented by the brown triangle) to TRs results in dissociation of CoR from the TR/RXR hetero-dimers and recruitment of CoA, inducing target gene transcription. B. Type 2 comprises the TRs-dependent actions of TH exerted through tethering to other chromatin proteins binding of T3-bound TRs to DNA. C. Type 3 TH actions comprises the nuclear TRs-independent TH actions exerted through direct or indirect binding to DNA. Especially, interaction of T3 with cytoplasmic TRs or with the truncated TRα isoforms p30 TRα1 at the plasma membrane stimulates signaling transduction that results in promotion of transcription; D. Type 4 comprises various nuclear TRs-independent TH actions as follows. D1. Binding of T3 to integrin αvβ3 activates the Src/PI3K/Akt pathway, leading to the shuttling of cytoplasmic TRα to the nucleus, promoting transcription. D2. Binding of T4 (represented by the green rhombus) and of T3 to integrin αvβ3 activates PI3K/Akt and MAPK)/ERK1/2 pathways via PLCP and KCα. Activated MAPK induces the sodium proton ex-changer (Na^+^/H^+^), increases activity of the sodium pump (Na, K-ATPase), and modulates intracellular protein trafficking of proteins, such as ERα and TRβ1, from the cytoplasm to nucleus, promoting the transcription. Activated PI3K transduces also signaling that promotes transcription. D3. Type 4 includes also the TH actions on polymerization of actin. D4. Type 4 includes the action of T3 as regulator of Crym. Abbreviations: A, sodium pump Na, K-ATPase; Akt/protein kinase B (PKB); AP, actin polymerization; CoA, coactivators; CoR, corepressors E, sodium proton exchanger (Na^+^/H^+^); ERK, extracellular signal-regulated kinase; P, proteins; PI3K, PKCα, protein kinase Cα; PLC, phospholipase C; PKGII, type II cGMP-dependent protein kinase; MAPK, mitogen-activated protein kinase; mTOR, mammalian target of rapamycin; NOS nitric oxide synthase; NP, nucleoproteins; RXR, retinoic acid X receptor; TR, thyroid hormone receptor; TRα1, TRalpha isoform 1; TRβ1, TRbeta isoform 1; TREs, thyroid response elements.

Type 4 includes also the TH actions on polymerization of actin and the action of T3 as allosteric regulator of Crym (or μ-crystallin), which is a NADP-regulated, T3-binding, cytoplasmic protein acting as ketamine reductase [72]. Actually, TH show weak binding affinity for several cytoplasmic proteins, but whether these proteins serve for transport and storage or initiate signaling events is unknown [42]. Figure 1 depicts the main signaling cascades implicated in the four types of TH actions.

## 4. The Contribution of TH to the “Hallmarks of Cancer”

The versatile role of TH in cancer is a complicated issue beyond the scope of the pre-sent review. Herein, we recapitulate the most representative data indicating the contribution of TH to the unique properties of cancer cells designated as “hallmarks of cancer” [73]. These “hallmarks of cancer” are signals of sustained proliferation, resistance to growth suppressors, evasion of programmed cell death, replicative immortality, sustained angiogenesis, promotion of invasion and metastasis, reprogramming of energy metabolism, and evasion from immune surveillance. Two enabling features have been suggested, namely, the genomic instability in cancer cells and the inflammatory state of premalignant and malignant lesions. In most cases, TH foster the acquisition of “hall-marks of cancer”. However, inhibitory effects of TH on these processes have also been described [74,75].

The pro-proliferative effect of TH on cancer cells is exerted through either the TRα or the integrin ανβ3. Critical signaling pathways that are regulated positively (PI3K/Akt, endoglin, sonic hedgehog [SHH], RAS-ERK, miR-21), negatively (ubiquitin-like with PHD and ring finger domains 1 [UHRF1]), or either positively or negatively (Wnt/b-catenin) by TH coordinate to modulate the expression of downstream targets implicated in cancer cell proliferation, such as E2F Transcription Factor 1 (E2F1), p21, c-myc, mTOR, and c-fos. Additionally, the pro-proliferative signaling that is initiated by the binding of TH to integrin ανβ3 can crosstalk with the pro-proliferative ERa signaling.

The pro-angiogenic effect of TH is exerted through the binding of T_4_ to integrin ανβ3, which activates the MAPK and PI3K/Akt pathways, resulting in modulation of expression of downstream targets, such as fibroblast growth factor 2 (FGF-2), vascular endothelial growth factor (VEGF), transforming growth factor alpha (TGFa), angiopoietin 2 (AGP-2), and hypoxia inducible factor 1 (HIF-1a).

The anti-apoptotic effect of TH on cancer cells is exerted mainly by physiological levels of T_4_ via signaling pathways, implicating the modulation of downstream targets, which in turn constitute critical components of the extrinsic and intrinsic pathways of apoptosis. Such downstream targets include: BcL-2 Associated X (BAX), BcLx-s, caspases, Rb, X-linked inhibitor of apoptosis (XIAP), cfos, cjun, p53, and p21.

Downstream targets of the T3-integrin ανβ3-MAPK pathway, such as matrix metalloproteinases (MMPs), thrombospondin 1 (TSP-1), and spondin-2, are known to enhance cancer cell migration. Interestingly, the role of T3 in invasion and metastasis can be dual, either promoting or inhibitory, as indicated by the paradigm of HCC. Treatment of HCC cells with T_3_ has been shown to enhance cancer cell migration and invasion by fostering overexpression of brain-specific serine protease 4 protein levels, which was associated with ERK1/2-C/EBPβ-VEGF cascade activation [76]. T_3_-induced reduction of expression of miR-17 and miR-130b [77,78], and overexpression of miR-21 [79] can foster the migration of cancer cells in HCC. However, T_3_ treatment has been shown to stimulate the overexpression of spondin-2, thereby abrogating the invasion and migration of cancer cells [80]. Additionally, T_3_ treatment can upregulate the expression of dickkopf Wnt (a fusion of the words wingless and integrated or int-1) signaling pathway inhibitor 4 (DKK4) protein, an antagonist of Wnt, in HepG2 TR-expressing cells [81], suggesting that the T_3_-TR-DKK4-Wnt/β-catenin cascade can inhibit metastasis [82].

Similarly, the effect of TH on the reprogramming of the metabolism of cancer cells is dual, either promoting or inhibitory. T_3_ can modulate the bioenergetics of cancer cells by increasing the mitochondrial metabolism and by regulating one of the isoforms of pyruvate kinase that is essential for the Warburg effect [73]. For instance, long-term treatment of MDA231 breast cancer cells with T3 has been shown to upregulate the glycolytic enzyme M2 isoform of the pyruvate kinase (PKM2), which promotes both the Warburg phenotype of cancer cells and the PGC-1α-mediated mitochondrial biogenesis [83]. On the other hand, T3 has been shown in animal models to exert an inhibitory effect on HCC progression by reverting the metabolic profile of HCC cells to that of a normal hepatocyte. Indeed, T3 can favor a switch from glycolysis to oxidative-dependent metabolism via TR-induced activation of KLF9—a transcription factor implicated also in cellular growth, development, differentiation, and inflammation [84].

Preliminary data, mainly nonclinical, indicate TH as critical determinants of immune response through activation of complex signaling pathways, affecting various immune cells [44]. Improved understanding of the immunobiology of dendritic cells (DCs)—the main antigen presenting cells—points to a pivotal role of TH in T cell responses. T3 is the main thyroid hormone that affects DCs, entering DCs more effectively than T4 through MCT10 and LAT2. Inside DCs, T4 is converted to T3 through DIO2, while T3 is inactivated to T2 through DIO3. T3 signaling in DCs is initiated at the cytoplasmic TRβ1, which is upregulated by NF-κB and activates the cytoplasmic Akt. In mice, in vitro and in vivo data show that T3 favors the phenotypic maturation of DCs through upregulating molecules of the major histocompatibility complex class II (MHCII), thereby promoting a pro-inflammatory cytokine phenotype, characterized by increased production of IL-12, IL-6, IL-23, IL-1β, and TGFβ1. Additionally, T3 can stimulate immune responses implicating Th1 cells, Th17 cells, IL-17-producing γδ T cells, and cytotoxic T cells. T3-conditioned DCs also increase CCR7 expression, facilitating their migration to lymph nodes, where they exert their antigen-presenting role [23].

Intriguingly, TH have a dual impact—both promoting and inhibitory—on the immunological responses. In mice, TH have been designated as “immune-endocrine checkpoints”, which act in a dual manner. On the one hand, T3 can induce downregulation of T regulatory (Treg) cells, expression of PD ligand 1 (PD-L1) on DCs, and expression of programmed cell death protein 1 (PD-1) on T cells. On the other hand, a physiological concentration of T_4_ (10-7M total; 10-10M free hormone) has been shown to increase PD ligand 1 (PD-L1) gene expression and PD-L1 protein levels in human breast cancer MDA-MB-231 cells and colon carcinoma HCT116 cells. In colon carcinoma HT-29 cells, T_4_ has been shown to increase the PD-L1 mRNA by 62% and the PD-L1 protein by 27%. The T_4_-induced PD-L1 accumulation was shown to implicate ERK1/2 activation [85].

Likewise, TH have a dual impact—both promoting and inhibitory—on the inflammatory responses. In mice, T3 has been shown to exert an anti-inflammatory effect mediated through TRβ1, attributed to (i) inhibition of differentiation of monocytes into macro-phages (in vitro), (ii) slight increase in peritoneal macrophage phagocytosis (in vivo), and (iii) switch of the phenotype of macrophages from M2 to M1 (in vitro) [86]. In humans, proinflammatory activities, such as the respiratory burst activity of polymorphonuclear leukocytes (PMNLs), can be induced by increased T_3_ levels [87].

**Figure 2:**
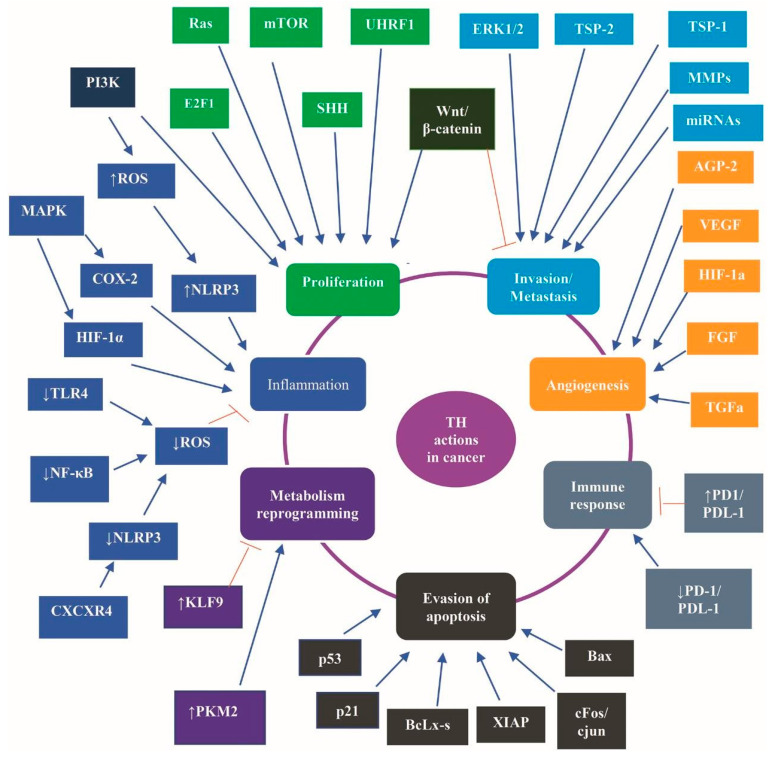
The contribution of TH to cancer hallmarks and the major involved signaling pathways. TH contributes to all the properties of cancer cells known as hallmarks of cancer, namely, to (i) proliferation, predominantly through SHH, UHRF1, mTOR, Ras, and E2F1; (ii) invasion/metastasis, predominantly through TSP-1, TSP-2, MMPs, miRNA, ERK1/2, and Wnt/β catenin; (iii) angiogenesis, predominantly through AGP-2 FGF VEGF, HIF-1α, and FGF; (iv) immune response, predominantly through PD-1 and PD-L1; (v) evasion of apoptosis, predominantly through BAX, p53, p21, cfos/cjun, XIAP, and BcLx-s; (vi) reprogramming of metabolism of cancer cells, predominantly through PKM2, KLF; and (vii) inflammation, predominantly through CXCR4, NF-κB, NLRP3, ROS, TLR4, HIF-1α, COX-2, MAPK, and PI3K. Abbreviations: AGP-2, angiopoietin 2, BAX, BcL-2 Associated X; COX-2, and cyclooxygenase 2; CXCR4, C-X-C motif chemokine receptor 4; E2F1, E2F Transcription Factor 1; ERK1/2, extracellular signal-regulated kinase 1/2; FGF, fibroblast growth factor 2; HIF-1α, hypoxia inducible factor 1; MAPK, mitogen-activated protein kinase; miRNA, microRNA; MMPs, matrix metalloproteinases; mTOR, mammalian tar-get of rapamycin; NF-κB, nuclear factor-κB; NLRP3, NLR Family Pyrin Domain Containing 3; TLR4, Toll Like Receptor 4; PD-1, programmed cell death protein 1; PD-L1, PD ligand 1; PI3K, phosphatidylinositol 3-kinase; PKM2, M2 isoform of the pyruvate kinase; ROS, reactive oxygen species; SHH, sonic hedgehog; TGFa, transforming growth factor alpha; TSP, Thrombospondin; UHRF1, ubiquitin-like with PHD and ring finger domains 1; VEGF, vascular endothelial growth factor; XIAP, X-linked inhibitor of apoptosis Wnt, a fusion of the words wingless and integrated or int-1.

Interestingly, a net effect of TH on immunological and inflammatory responses has been described, depending on concentration and availability of circulating and intracellular TH. A core component of this net effect is the metabolic status that modulates the activation and stability of NLR Family Pyrin Domain Containing 3 (NLRP3) inflammasome—an intracellular complex that regulates the innate immune activity through modulation of production of pro-inflammatory cytokines [44]. Elevated levels of T4 through the T4-integrin αvβ3-PI3K-Akt signaling cascade induce a robust production of Reactive Oxygen Species (ROS), which trigger NLRP3 inflammasome. Additionally, the T4-integrin αvβ3-MAPKs cascade stimulates the expression of hypoxia inducible factor 1 (HIF-1α) and cyclooxygenase 2 (COX-2) to foster NLRP3 inflammasome assembly and stability. Physiological levels of T3 favor anti-inflammatory responses, bactericidal activity, and phagocytosis. T3 decreases ROS levels through the T3-TRs-TREs complex-induced downregulation of Toll Like Receptor 4 (TLR4), NF-κB, NLRP3, pro-IL-1β, and pro-IL-18 and several miRNAs, such as miR-31, miR-155, and miR-222. Moreover, the T3-TRs-TREs complex inhibits the assembly of NLRP3 inflammasome through upregulation of miR-30, miR-133, and miR-144, which target Fasl, Ilk, Serpine1, hepatocyte growth factor (HGF), Beta secretase 1 (Bace 1), and C-X-C motif chemokine receptor 4 (CXCR4) [44]. TH have been shown to increase the sensitivity of natural killer cells (NK) to IFN-γ in murine cells [88], while having a dual impact—either promoting or inhibitory—on NK activity in humans, depending on TH concentration [23,89]. Overall, the net effect of TH on immunological and inflammatory responses depends on the concentration and the availability of circulating and intracellular TH and on the metabolic status that modulates the NLRP3 inflammasome activation and stability [44]. Figure 2 illustrates the contribution of TH to cancer hallmarks and the major involved signaling pathways.

An integrin αvβ3-mediated TH action in tumor stroma formation—a core component of tumor microenvironment—has been reported in a subcutaneous HCC xenograft model. T3 or T4 increased the recruitment and the invasion of mesenchymal stem cells (MSCs) into tumors of hyperthyroid mice compared to tumors of euthyroid and hypothyroid mice and stimulated the expression of genes related to cancer-associated fibroblast-like differentiation. In primary human bone marrow-derived MSCs, treatment with T3 or T4 in the presence of HCC-conditioned medium stimulated the expression of genes related to cancer-associated fibroblast-like differentiation. Treatment with tetrac eliminated MSCs recruitment almost completely [90].

Importantly, a key determinant of the dual role of TH in cancer is the T3-induced premature senescence. In mouse embryonic fibroblasts (MEFs), T3 has been shown to rapidly activate the ataxia telangiectasia mutated (ATM)/adenosine monophosphate-activated protein kinase (PRKAA) signal transduction, leading to recruitment of NRF1 (nuclear respiratory factor 1) and of TRβ to promoters of key genes of mitochondrial respiration. The resultant oxidative stress can induce double-strand break, eliciting a persistent DNA damage response, which in turn stimulates senescence [91]. The latter is a complex, dynamic, and interactive status of stable proliferative arrest triggered by various stimuli, such as oncogenic cellular stress, resulting in tumor suppressive or pro-oncogenic effects on adjacent cancer cells and other components of tumor microenvironment (e.g., stroma, vasculature, and immune system). In fact, senescence has been designated as “the true power behind the throne”, fostering several “hallmarks of cancer” [92].

## 5. Insights into the TH–Lung Cancer Association Gained over the Last Three Decades

### 5.1. Insights from Nonclinical Data

Over the last three decades, an increasing amount of nonclinical data have indicated the divergent effects of TH in lung cancer.

In 1991, Kinoshita et al., working on a murine model of lung cancer, demonstrated that a hyperthyroid status characterized by elevated T3 and reduced T4 levels, induced by subcutaneous injections of T3, significantly inhibited the spontaneous pulmonary metastases of Lewis lung carcinoma (3LL), prolonging the survival of mice. On the contrary, treatment with T4 induced a hyperthyroid status with elevated T3 and T4 levels, which enhanced primary tumor growth and development of pulmonary metastases of 3LL. Consistent with this finding, methimazole-induced hypothyroidism characterized by reduced T3 and T4 levels was shown to suppress primary and metastatic tumor growth, resulting in prolongation of survival. Additionally, distinct interventions in the TH status were shown to have a differential impact on the host immune surveillance. Injections of T3 and T4 for 4 weeks reinforced the cytotoxicity of alveolar macrophages against 3LL tumor cells. Treatment with T4 or methimazole attenuated the NK activities of spleen cells compared to control mice, but T3 had no impact on NK activities [93].

TH, especially T4, were shown to favor tumor growth in a mouse model of lung adenocarcinoma by Theodossiou et al. The authors intervened in the TH status of athymic nude mice through either administration of oral propylthiouracil (PTU) or combination of PTU and thyroxine. Subsequently, the animals were inoculated with lung adenocarcinoma cells. Tumor growth was attenuated in mice with PTU-induced hypothyroidism compared to tumor growth in mice with hyperthyroid T4 levels induced by combination of PTU and thyroxine, the latter being similar to the tumor growth in control euthyroid animals. In hypothyroid mice, restoration of euthyroidism after withdrawal of oral PTU allowed tumor growth. Furthermore, in vitro and in vivo data excluded a direct inhibitory effect of PTU on tumor growth; thus, the hypothyroid status per se was credited with attenuation of tumor growth [94].

The report of the implication of decreased TRβ expression via hypermethylation of the promoter of the corresponding gene in human breast cancer provided the rationale for the hypothesis that the role of TH in lung cancer is mediated by aberrant TRβ function [95]. To evaluate this hypothesis, Iwasaki et al. explored the expression, mutation, and promoter methylation of TRβ1 in 18 small cell lung cancer (SCLC) and 29 non-small cell lung cancer (NSCLC) cell lines by reverse-transcription polymerase chain reaction (RT-PCR), direct sequencing, or methylation-specific PCR, using four non-tumor human bronchial epithelial cells as normal controls. This study demonstrated: (i) absence of TRβ1 ex-pression in 61% of SCLCs and 48% of NSCLCs, (ii) TRβ1 promoter methylation in 67% of SCLCs and 45% of NSCLCs, and (iii) no TRβ1 somatic mutation. TRβ1 methylation status was significantly correlated with loss of TRβ1 expression. Treatment with 5-aza-2-deoxy-cyti-dine and/or trichostatin-A in four cell lines resulted in restoration of TRβ1 expression [95]. Taken together, these findings indicated TRβ1 as a tumor suppressor likely implicated in lung carcinogenesis [95].

Meng et al. demonstrated that interaction of TH with integrin αvβ3 can induce proliferation of human NCI-H522 NSCLC cells and NCI-H510A SCLC cells through a crosstalk between integrin αvβ3-mediated and ERa-mediated signaling in a concentration-de-pendent manner. T4 at a physiologic concentration (10−7 M) and T3 at a supraphysiologic concentration significantly increased proliferating cell nuclear antigen (PCNA) abundance in both NCI-H522 cells and NCI-H510A cells. Anti-integrin αvβ3 antibodies, but not anti-integrin αvβ5 antibodies, were demonstrated to abrogate the T4-induced PCNA accumulation in both NCI-H522 cells and NCI-H510A cells, indicating integrin αvβ3 as mediator of T4-induced PCNA accumulation in lung cancer cells. Similarly, tetrac was shown to abrogate the TH-induced PCNA accumulation and the ERK1/2 activation in NCI-H522 and NCI-H510A cells by inhibiting the interaction of TH with integrin αvβ3. Additionally, T_4_ was demonstrated to cause nuclear accumulation of phosphorylated ERα in NCI-H522 cells, an action inhibited by co-incubation of cells with T_4_ and tetrac. ICI 182,780 (ICI), an ER antagonist, inhibited ERα phosphorylation, ERK1/2 activation, and NCI-H522 cells proliferation, while the impact of ICI on the action of T3 was minimal. Taken together, T4-induced lung cancer cell proliferation initiated at integrin αvβ3 was shown to be mediated by activated ERα, whereas T3-induced cell proliferation was independent of ERα phosphorylation [96].

Mousa et al. demonstrated the proliferative effect of TH and the anti-proliferative and anti-angiogenic action of tetrac-NP in three models of NSCLC: (a) cultured human NSCLC H1299 cells in vitro, (b) tumor cell implants in the CAM model, and (c) xenografts in the nude mouse. In vitro, TH-induced cell proliferation was inhibited by an antibody blocking integrin αvβ3, by unmodified tetrac and by tetrac-NP. Additionally, the TH-induced proliferation of NSCLC cells was inhibited by pharmacologic blockade of the MAPK pathway. In H1299 cells grown in the CAM model, tetrac and tetrac-NP attenuated tumor growth and tumor-related angiogenesis; additionally, both agents precluded chick embryo mortality. Tetrac or tetrac-NP administered intraperitoneally every 2 days in xenograft tumor models established by subcutaneous inoculation of H1299 cells in nude mice significantly suppressed tumor growth and angiogenesis [97].

An interesting mechanism through which TH modulate gene expression in human NSCLC cells implicates the integrin αv monomer, as indicated by the study of Lin et al. [98]. Albeit conventionally considered a cell surface protein, the integrin αv monomer can be internalized and translocated in the nuclear of human NSCLC cells through T4-medi-ated mechanisms. In TH-treated lung cancer cells, the interaction of integrin αv monomer with two activators of transcription (p300 and phosphorylated [p] STAT1) was shown to be involved in the genomic actions of TH. Especially, the nuclear p300 complex that includes pERK1/2 and integrin αv monomer was shown to regulate TH target genes critical for tumor cell biology, such as COX-2, hypoxia inducible factor 1 alpha (HIF-1α), and ERα. The integrin αv monomer formed also complexes with NCoR and SMRT to modify the transcriptional activity of several members of the nuclear superfamily of hormone receptors, including TR. The interaction of integrin αv monomer with pERK1/2 and with nuclear coactivators and corepressors was prevented by integrin β3 siRNA, but not by scRNA, indicating the necessity of the existence of intact heterodimer at the point of internalization of the integrin. The role of integrin αv monomer in the nucleus was at-tributed to conserved sequence(s) in αv monomer that recognize specific domains of p300 related to transcription, facilitating the trafficking of T4-activated ERK1/2 into nucleus and its interaction with the p300 complex [98].

Carmona-Cortés et al. evaluated the effects of the interaction of TH with nitric oxide (NO) on tumor development and angiogenesis in a murine model of implanted Lewis’s carcinoma. T4 was shown to induce parallel increases in tumor weight (TW) and angio-genesis. The NO inhibitor L-NAME reduced TW and angiogenesis in control, hyperthyroid, and hypothyroid mice, whereas no variable was influenced by inducible form of NOS (iNOS) inhibitor aminoguanidine (AG). Tetrac attenuated TW in normal and T4-treated mice, whereas it did not inhibit angiogenesis in T4-treated mice. TW was negatively correlated with AP activity in tumors from control hyperthyroid and hypothyroid groups, and an inverse relationship was observed between TW and AP activities in tetrac-treated mice. T4 reinforced TW and NO-mediated angiogenesis, but T4-induced carcinogenesis required activation of integrin αvß3. NO blockade decreased TW, irrespectively of the thyroid status. Taken together, this study demonstrated that TH increased TW via, at least partially, negatively modulating the AP activity in the tumor, and that inhibition of the membrane TR at αvß3 integrin decreases TW via, at least partially, enhanced AP activity [99].

Recently, the effect of T_4_ on lung cancer growth was studied in an orthotopic mouse model by Latteyer et al. In this study, murine Lewis lung carcinoma (3LL) cells, stably transfected with luciferase, were injected into mouse lungs. Tumor growth in untreated mice was compared to that in hypothyroid mice, and hypothyroid mice treated with T_3_ or T_4_ with or without tetrac. This study demonstrated that tumor growth was reduced in hypothyroidism and was increased by T_4_ treatment. Intriguingly, only T_4_ treatment pro-moted tumor growth, an effect that was inhibited by tetrac. Tumor weight and neoangiogenesis were also significantly increased only in T_4_-treated mice, but T4 did not influence ανβ3 expression. A direct effect of T4 on proliferation of 3LL cells was excluded in vitro [100]. Taken together, in this study, the tumor-promoting effect of T4 was mediated by integrin ανβ3 and was attributed to increased neoangiogenesis rather than direct stimulation of 3LL cells.

Overall, according to nonclinical data, TH signaling is implicated in lung cancer progression, but its effect can be either promoting or inhibitory depending, at least partially, on TH type (T3 or T4), TR type and isoform, TH concentration, and activated molecular pathways. However, it is acknowledged that most nonclinical data are derived from ani-mal models, and thus, more human nonclinical data are needed. Table 2 summarizes the nonclinical studies addressing the TH-lung cancer association.

### 5.2. Insights from Clinical Data

The hallmark case report of Hercbergs and Leith in 1993, pointing to the anticancer potential of hypothyroidism in lung cancer in the clinical setting [6], launched an evolving field of research. In this case report, the authors described the remission of metastatic NSCLC, previously treated with chemotherapy and radiotherapy, after recovery of myxedema coma induced by amiodarone in a 69-year-old male patient [6]. The myxedema coma was initially treated with high doses of levothyroxine (LT4). Subsequently, the patient received daily LT4 at maintenance doses, as well as digoxin and diuretics for cardiac disease, and preferred cessation of chemotherapy. Surprisingly, no recurrence or progression of NSCLC was documented during a 4-year follow-up after myxedema coma. The patient died of myocardial infarction. Given that the clinical remission of NSCLC was co-incident with the recovery of myxedema coma, the authors hypothesized that it was precipitated by severe T3 deficiency and suggested the inhibition of the proapoptotic role of T3 as underlying mechanism [6].

A retrospective case control study assessing the occurrence of primary hypothyroid-ism among 1979 patients with NSCLC and SCLC (stages I-IV) revealed 85 cases of hypothyroidism (4.2%). The female to male ratio for hypothyroidism was 3.35:1. The number of patients with hypothyroidism differed among distinct disease stages, with 86 patients, 46 patients, 19 patients, and 19 patients in stages III, IV, I, and II, respectively. Improved median survival for disease stages I-IV was observed in the hypothyroid group compared to the euthyroid group (14.5 months versus 11.1 months, respectively; *p* = 0.014). Especially, for stage IV, median survival was 11 months in the hypothyroid group versus 5 months in the euthyroid group (*p* = 0.0018). Taken together, primary hypothyroidism was suggested as significant prognostic factor for a favorable clinical outcome of lung cancer [101].

Assessment of the pattern of HPT axis function in the setting of a 24 h sleep-wake schedule in 11 healthy participants (age of 35–53 years-old) and 9 patients with NSCLC (age of 43–63 years-old) by Mazzoccoli et al. demonstrated that, compared to healthy participants, NSCLC patients had lower 24 h means TSH levels, but higher 24 h means TRH and fT4 levels. Nevertheless, both groups of patients experienced similar synchronization of the main body clock to the 24 h environmental light-dark cycle, resulting in a prominent circadian rhythm of TSH with peaks near midnight [102].

A prospective study assessing the risk of cancer, including lung cancer, in 29,691 individuals revealed development of cancer in 2511 individuals during a median follow-up of 9 years. TSH levels below the lower reference range (<0.50 mU/L), suggestive of hyperthyroidism, were correlated with higher risk for any cancer type compared to any other TSH levels including the highest TSH levels suggestive of hypothyroidism. Individuals with TSH levels <0.50 mU/L had a 34% higher cancer risk compared to individuals with TSH within the reference group (0.50–1.4 mU/L) (adjusted hazard ratio [HR], 1.34; 95% confidence interval [CI], 1.06–1.69). Compared to the reference group, the adjusted HR particularly for lung cancer was 2.34 (95% CI, 1.24–4.40). Adjustment for smoking only moderately attenuated this association [HR, 2.60 (unadjusted) versus 2.34 (adjusted)]. A separate analysis excluding the first 2 years of follow-up to eliminate a potential bias of impact of preclinical cancer on TSH levels revealed even stronger association of hyperthyroidism with lung cancer (HR, 2.91, 95% CI, 1.49–5.70). Individuals with subclinical hyperthyroidism were at higher risk for lung cancer compared to the euthyroid reference group (TSH, 0.50–1.4 mU/L) with a HR of 2.55 (95% CI, 1.22–5.35). Overt hyperthyroidism showed stronger association with lung cancer risk compared to subclinical hyperthyroidism, but a precise estimation was not feasible due to small number of patients [103].

A prospective population-based cohort study of 10,318 participants with available baseline evaluation of fT_4_ and/or TSH levels demonstrated a significant association be-tween higher fT_4_ levels and higher risk of lung cancer (HR, 2.33; 95% CI, 1.39–3.92), which maintained its significance after excluding administration of thyroid-altering medication. Compared to fT4 levels of the lowest tertile (0.12–1.14 ng/dL), fT_4_ levels of the highest tertile (1.29–4.73 ng/dL) were correlated with a 1.79-fold increased risk of lung cancer (*p* for trend <0.05). No association between TSH levels and cancer risk was observed. The association between thyroid function and cancer risk did not differ according to sex or age [104].

Overall, preliminary clinical data indicate an anticancer potential of hypothyroidism in lung cancer as opposed to the tumor-promoting potential of hyperthyroidism.

On the other hand, in a prospective cohort study of a community-dwelling population of 3649 persons in Western Australia, 600 individuals were diagnosed with non-skin cancer during a 20-year follow-up. Forty-one patients had lung cancer, but no association of TSH, fT4, or anti-TPO antibodies (anti-TPOAbs) with lung cancer was observed [105].

Iwasaki et al. investigated the correlation between epigenetic inactivation of TRβ1 through aberrant methylation and clinicopathological parameters or mutations of KRAS and EGFR in 116 NSCLC surgical specimens. TRβ1 methylation was detected in 47% of NSCLC surgical specimens, while methylation demonstrated no significant association with any clinicopathological parameters or mutations of KRAS and EGFR [95].

Little is known about the correlation of administration of LT4, which is the mainstay treatment of hypothyroidism, with risk of lung cancer. A study investigating the correlation of sales of LT_4_ in 2009 with the prevalence of several cancer types including lung cancer in women of 30–84 years-old in 18 Italian regions during 2010 demonstrated a significant correlation between LT4 administration and lung cancer (*p* < 0.05) corrected for smoking and age. None of the other cancer types studied (breast, colorectal, and gastric cancers) showed significant correlation with LT_4_ sales. The authors postulated that LT4 may contribute to lung carcinogenesis via triggering oxidative stress. However, it was hypothesized that the hypothyroidism for which LT4 was administered could be implicated in lung carcinogenesis [106,107]. Table 3 depicts some representative studies addressing the TH-lung cancer association.

In the era of precision oncology, a novel aspect of the TH-lung cancer association is the thyroid impairment related to immune checkpoint inhibitors (ICPi) and tyrosine kinase inhibitors (TKI), which have revolutionized the landscape of NSCLC therapeutics [12,107,108,109,110,111,112]. Detailed discussion of this issue is beyond the scope of the present review. Briefly, thyroid disorders related to ICPi and TKI are a common adverse event, potentially related with favorable prognosis. Table 4 depicts some representative studies concerning the impact of ICPi [113,114,115,116] and TKI [117,118,119,120,121,122] on thyroid function to highlight the need for thyroid function monitoring in patients receiving these agents.

## 6. Association of Non-Thyroidal Illness Syndrome (NTIS) with Lung Cancer

The non-thyroidal illness syndrome (NITS) is strongly associated with lung cancer. NTIS, also known as sick euthyroid syndrome, is a status of altered TH encountered in seriously ill or starving patients, characterized by low fT3, usually elevated rT3, normal or low TSH levels and, in case of prolonged duration and increasing severity of the illness, decreased T_3_ and T_4_ levels. fT4 levels can be low, normal, or elevated, depending on the timing of the measurement and the assay. TSH can be reduced, but not less than 0.05 μU/mL. In any case, TSH is inappropriately low for the serum T_4_ and T_3_ levels. During recovery, TSH transiently increases above normal.

Although the pathophysiology of NTIS remains elusive, it may implicate suppression of TRH release, reduced turnover of T3 and T4, reduced generation of T3 in liver, increased formation of rT3, and tissue-specific down-regulation of DIO, transporters, and TRs. Additionally, cytokines are considered key determinants of NTIS, inhibiting all steps of synthesis and secretion of TH, as well as suppressing TSH. Tissue TH levels are reduced, resulting in tissue hypothyroidism, which is not clinically evident because of short duration. The interpretation of the syndrome is debated. Certain researchers consider NTIS as adaptive mechanism of HPT axis in response to acute or chronic illness, which should not be treated. Others juxtapose that TH replacement is appropriate, not disadvantageous, and occasionally advantageous [123,124].

The first clinical evidence of NTIS in lung cancer patients was reported as early as in 1978 by Ratcliff et al. The authors assessed the thyroid function at the time of initial diagnosis of lung cancer in 204 lung cancer patients compared to that of age and sex-matched patients with non-malignant lung disease. Thyroid disorders were observed in 67 patients (33%) and manifested principally as low T3 levels with no clinical or biochemical evidence of hypothyroidism. Other thyroid disorders were primary hypothyroidism, low T4 levels and free thyroxine index (FTI: product of the measured T_4_ level and the tri-iodothyronine uptake) with normal TSH levels, moderately increased TSH with normal TSH levels, and increased FTI with or without increased T4 levels. Taken together, TH metabolism in lung cancer tended towards reduced T3 levels with significantly increased T4/T3 ratios and modestly increased rT3 levels, pointing to decreased 5′-mono-deiodination of T4. Six-month mortality was higher in lung cancer patients with low T3 levels compared to matched lung cancer patients with normal T3 levels (49% versus 27%, respectively). No significant difference in TH or TSH levels between patients with apparently localized disease (*n* = 103) and patients with extrathoracic metastases (*n* = 101) was observed. Patients with lung cancer of any histological type had higher T4/T3 ratio than controls, but the difference was proved statistically significant only for the small-cell anaplastic group [125].

NTIS was suggested as predictor of unfavorable prognosis in NSCLC patients in a prospective study enrolling 80 patients with newly diagnosed NSCLC. In this study, the incidence of NTIS was 35%, with NTIS being more frequent in stage III (26%) and stage IV (62%) disease. NTIS was correlated positively with disease stage and weight loss. Mean follow-up time was 13.3 ± 7 months. Mean survival time was 9.2 and 15.2 months for patients with and without NTIS, respectively. This difference in survival was significant in both univariate analysis (*p* = 0.0002) and multivariate analysis adjusted for histological type and stage of tumor, body mass index (BMI), and ratio of weight loss (*p* = 0.04). No difference in general survival rate (*p* = 0.8) was observed between cases with NTIS type 1 (only fT3 decreased) and NTIS type 2 (fT3 and fT4 decreased) [126].

Another prospective study enrolling 120 patients (71 with NSCLC and 49 with SCLC) demonstrated that NTIS—identified in 42% of NSCLC patients and 44% of SCLC patients—is correlated with aggressive cancer behavior and decreased survival in both NSCLC and SCLC, compared to no NTIS [127]. NTIS incidence was significantly higher in stage IV (54%) compared to stage II (38%) NSCLC (*p* = 0.032) and in extensive disease (59%) compared to limited disease (0%) of SCLC (*p* = 0.0002). NTIS type 1 showed similar frequency among NSCLC and SCLC patients, and the same was observed for type 2 NITS. According to a Cox proportional hazard model adjusting for factors significant in the univariate analysis, NTIS remained an independent risk factor for mortality in all cancer patients (HR, 4; 95% CI 2.3–6.9; *p* = 0.000). Collectively, NTIS was suggested as independent prognostic factor for lung cancer, but this result needs to be confirmed in future studies with greater sample sizes [127].

## 7. Current Challenges and Future Perspectives

Given the complexity of the TH-lung cancer association, the prioritization of relevant research requests is daunting, yet critical. The overarching aim is to accomplish a molecularly-based tailored approach to TH–lung cancer association. To this end, it is essential to overcome current challenges arising from the three queries that set the framework of the present review, as suggested in the introduction of the present review.

The challenges arising from the first two queries (How to stratify patients with TH-sensitive lung tumors who may benefit from interventions in TH status? How is deter-mined whether TH promote or inhibit lung cancer progression?) may be overcome with advances in the understanding of the molecular background of the contribution of TH to the hallmarks of cancer, taking into consideration the heterogeneity of lung cancer in terms of histology, molecular background, and biological behavior [128]. Delving into the biology of lung tumors, of TH signaling, and of the interrelationship thereof could lead to identification and validation of tumor-specific and patient-specific biomarkers of sensitivity of lung tumors to TH to enable stratification of patients who are expected to benefit from anticancer interventions in TH status. It could also lead to identification of patient-specific and/or cancer-specific factors that determine the effect of TH on cancer outcome. The transcriptional profiling—a promising tool to stratify cancer patients and guide anti-cancer treatment—may facilitate the comprehensive characterization of the expression level of the TH-related pathways and genes involved in lung cancer progression [129].

Further research is needed to counteract current obstacles to validation of biomarkers of the TH-lung cancer association. First, harmonization and standardization of key platforms could resolve challenging issues, such as assay variability, different platforms, and lack of reference standards [130]. Second, due to inherent difficulties in tumor sampling, the investigation of molecular biomarkers in peripheral blood is a more tangible perspective. Third, innovative technologies, including multicolor flow and mass cytometry, whole transcriptome sequencing, epigenetic analysis, and multianalyte serum immuno-assays are expected to reveal “signatures” of the TH-lung cancer association to be evaluated both retrospectively and prospectively.

A hindrance to evaluation of the effect of TH on cancer outcome is that the circulating TH levels may not reflect the intratissue and intracellular TH levels. The latter are influenced by several factors, such as (i) the TH blood transporters and membrane carriers, (ii) the interaction of extracellular TH with membrane receptors, (iii) the tissue-specific and cell type-specific expression of distinct DIO, (iv) the tissue-specific and cell type-specific expression and/or activity of distinct TRs isoforms, and (v) the clinical relevance of the derivative metabolites of TH [131]. Accurate assessment of intratissue and intracellular TH status can be achieved via exploration of the metabolome signature of TH status [132,133].

Future clinical studies assessing the contribution of TH to lung cancer outcome should address current methodological pitfalls, such as (i) evaluation of fT4 levels only in case of TSH values suggestive of hypothyroidism or hyperthyroidism, (ii) absence of adjustment of risk estimates for potential confounders, and (iii) paucity of information regarding the use of thyroid medication.

The road to answering the third question suggested in the introduction of the present review (How to leverage the antitumor effects of TH and/or abrogate the tumor-promoting effects of TH in the clinical setting?) is long and winding. An interesting perspective is to further explore two interventions in TH status with presumed anticancer potential: (i) the induction of euthyroid hypothyroxinemia via antithyroid drugs and concomitant administration of LT3 [63,134] and (ii) the administration of LT3 instead of LT4 as TH replacement treatment [63]. The rationale behind these interventions is the knowledge that the tumor-promoting effects of TH are exerted principally through the T4-integrin αvβ3 interaction as opposed to the pro-metabolic effects of Liothyronine (LT3).

In the same context, LT3 is of great interest for four additional reasons. First, very recently, Zhou et al. suggested that LT3 could be repositioned for cancer immunotherapy based on its ability to block the T Cell Immunoreceptor with Ig and ITIM domains (TIGIT)/poliovirus receptor (PVR) interaction—an emerging immune checkpoint system [135]. Second, the ability of LT3 to induce phenotypic and functional activation of DCs [23] could potentiate the promising dendritic cell-based immunotherapy in lung cancer [136]. Third, preliminary in vitro data have suggested that pretreatment with LT3 or administration of LT3 during chemotherapy could reinforce the efficacy of chemotherapy—a promising strategy known as “choriocarcinoma-mimic chemotherapy”, or “neo-endocrinochemotherapy”. This strategy capitalizes on the knowledge that certain choriocarcinoma and testicular tumors can be cured by chemotherapy alone thanks to elevated levels of human chorionic gonadotropin (hCG) with a TSH-like effect, leading to hyperthyroid status, which in turn potentiates the efficacy of chemotherapy [137]. However, a limitation to the use of LT3 is its short half-life and the risk of complications affecting mainly the cardiovascular system and the mineral metabolism in case of iatrogenic hyperthyroid-ism due to LT3 overdosing. To this end, the characterization of the LT3 pharmacokinetics is an essential step to design appropriate schemes of LT3 dosage [138].

Although the pharmaceutical industry has conducted several efforts to develop effective thyromimetics and/or TH antagonists, this enterprise remains a critical challenge. The identification of TH derivatives that could uncouple the therapeutic actions of TH from deleterious effects of a thyrotoxic state (e.g., tachycardia, arrhythmia, muscle catabolism, reduced bone mineralization, mood disorder) has been long pursued. The varying tissue distribution of specific TRs isoforms has inspired the development of TRβ selective thyromimetics, but their clinical application is hampered by side effects. The Sobetirome (GC-1) and, subsequently, the Eprotirome (KB2115) were the first TRβ selective thyromimetics with multiple therapeutic applications, including an anticancer potential, to enter clinical trials; however, Phase 1 and Phase 2–3 clinical trials, respectively, were terminated early due to toxicity [139]. Currently, the interest for this area has been rekindled by the design of “tissue-selective prodrugs” capable of providing the active compound at the site of action [25].

Although the 3,5-T2 was initially conceived as thyromimetic agent [29,30,31,32], it was proved an ambiguous, Janus-faced TH metabolite with beneficial and adverse bio-logical effects. More translational research is needed to allow the development of 3,5-T2-related agents [140].

As regards the development of antagonists of TH, further clinical studies are needed to explore the in vitro and in vivo anticancer actions of tetrac and NDAT. Such actions include (i) inhibition of proliferation, angiogenesis, and metastasis of cancer cells; (ii) pro-motion of apoptosis of cancer cells; (iii) enhancement of immune surveillance; (iv) repair of double-strand DNA breaks; (v) chemosensitization; and (vi) radiosensitization [62,67,68,69,70,71,141]. Especially in lung cancer, attenuation of the T4–integrin αvβ3–PI3K/Akt signaling cascade could abrogate cancer progression through downregulation of NLRP3 gene expression, considering that NLRP3 inflammasomes in lung cancer have been demonstrated to promote tumor growth, proliferation, invasion, and metastasis [142]. Compared to unmodified tetrac, NDAT is a more potent TH antagonist at αvβ3 and regulates a broader range of cancer-related genes [66]. Additionally, the ability of NDAT to attenuate the oncogenic signaling driven by Ras mutations in human breast cancer cells [143] and the T4-induced expression of PDL-1 in human breast cancer and colon cancer cells [85] should be investigated in the setting of lung cancer.

Resveratrol—a natural polyphenolic stilbene isolated from various plants, foods, and beverages credited with anticancer properties—has been shown to mimic tetrac, competing with TR at integrin αvβ3 [5]. It would be of interest to investigate whether this action contributes to the promising anticancer properties of resveratrol and, especially, of the most potent and privileged resveratrol-based compounds that have been developed recently [144].

**Figure 3:**
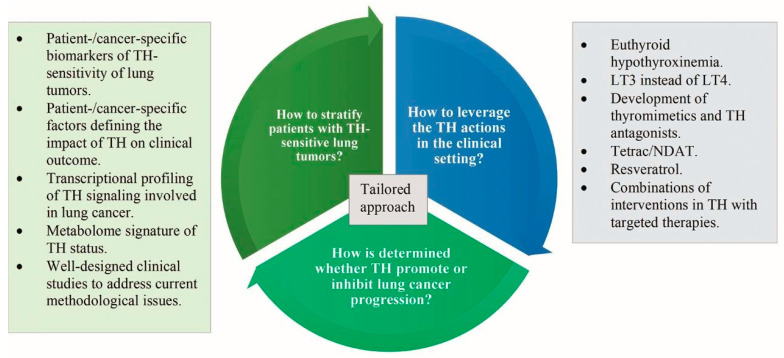
The pending queries and the main corresponding research priorities relevant to a tailored approach to the TH–lung cancer association. The tertiles of the pie depict the three pending queries, and the corresponding rectangles depict the corresponding research priorities. To stratify patients with TH-sensitive lung tumors and to clarify the dual role of TH in lung cancer, the research priorities are similar and include identification of patient-/cancer-specific biomarkers/risk factors, transcriptional profiling of TH signaling cross-talking with oncogenic signaling, exploration of the metabolome signature of TH status to assess precisely the intracellular and intratissue TH status, and well-designed clinical studies. To leverage the TH-lung cancer association in the clinical setting, the research priorities include exploration of interventions in TH as anticancer strategies, such as euthyroid hypothyroxinemia or administration of LT3 instead of LT4 to treat hypothyroidism; development of thyromimetics and TH antagonists; exploration of the anticancer potential of tetrac, NDAT, and resveratrol; and establishment of combinations of interventions in TH with targeted therapies. Abbreviations: LT3, liothyronine; LT4, levothyroxine; NDAT, nano-diamino-tetrac; TH, thyroid hormones.

In the era of precision medicine, the path forward for the exploitation of the TH-lung cancer association is to integrate an anticancer intervention in TH status (if exists) with targeted therapies and immunotherapy. In this context, more research should address the interactions of TH signaling with targetable tumor-promoting signaling pathways, such as the PI3K/Akt, the PD-1/PD-L1, the TIGIT/PVR, and the Ras mutations-driven cascades [145,146,147]. Figure 3 illustrates the pending queries and the main corresponding research priorities relevant to a tailored approach to the TH–lung cancer association

Last, but not least, the TH–lung cancer association in the context of the ongoing corona-virus disease 2019 (COVID-19) pandemic constitutes a research priority for many reasons. First, the thyroid gland is well implicated in the COVID-19 disease [124,148,149]. Second, low fT3 levels—often encountered in lung cancer patients with NTIS—have been indicated as independent predictor of the severity of COVID-19 disease [124,150]. Third, cancer [151,152,153,154,155], including lung cancer [153,154,155], has been recognized as risk factor for increased severity of COVID-19. Fourth, more research and long-term epidemiological surveillance are essential to answer whether the interplay between TH and COVID-19 can contribute to lung carcinogenesis or aggravate a preexisting lung malignancy [156,157]. In fact, there is ample epidemiological evidence concerning the COVID-19–thyroid association and the COVID-19–cancer association, while the relevant molecular mechanisms are currently under research. The authors of the present review have extensively discussed these topics elsewhere [158]. Briefly, the molecular background of the COVID-19–thyroid association includes a direct injury of the thyroid gland through inflammatory and immunological reactions and/or damage of the HPT axis either directly or indirectly. The currently available data concerning the molecular link between COVID-19 and cancer point to the assumption that cancer and COVID-19 meet at the crossroad of “inflammaging”, a state of aberrant systemic inflammation due to cytokine dysregulation ascribed to remodeling of immune system, and “immunosenescence”, a state of compromised immune system function. Both states are promoted by comorbidities/risk factors common between cancer and COVID-19, namely, aging, obesity, oxidative stress, and metabolic syndrome [159]. With 7964 publications regarding the thyroid disorders–COVID-19 association and 7355 publications regarding the COVID-19–lung cancer association in the Lit COVID, as of 21 December 2021 [160], the presumed interplay between COVID-19 and thyroid and lung cancer is not negligible. More translational research is needed to unravel the molecular background of this interplay.

## 8. Conclusions

Exemplifying the long-pursued TH–cancer association, TH signaling is interrelated with lung cancer in a dual manner, either promoting or inhibitory. Deciphering the crosstalk between TH signaling and oncogenic signaling in lung cancer may enable the integration of interventions in TH status with anticancer potential into oncologists’ armamentarium. However, several pending issues need to be resolved to recommend a tailored intervention in the status of TH as a strategy to battle lung cancer consistent with the principle “first, do no harm”.

## Figures and Tables

**Table 1:** The main properties of distinct deiodinases.

Property	DIO1	DIO2	DIO3
Reaction catalyzed	Deiodination of both outer and inner rings of iodothyronines	Deiodination of the outer, phenolic ring	Deiodination of the inner tyrosine ring
Localization	Plasma membrane	Endoplasmic reticulum	Plasma membrane
Tissue distribution	Thyroid gland, liver, pituitary, kidney	Thyroid gland, brain, pituitary skeletal muscle, brown adipose tissue	Brain, pancreas, fetal tissues, placenta
Action	➢ Transformation of T4 to T3 (K_m_ in the range of 1–0 μm)	➢ Transformation of T4 to T3 (K_m_ in the range of 1–4 nm) ➢ Transformation of rT3 to T2	➢ Transformation of T3 to T2 ➢ Transformation of T4 to rT3
➢ Transformation of rT3 to T2			
Substrates ^a^	rT3 > T4 >T3	T4 > rT3	T3 > T4
Hypothyroidism	↓	↑	↓
Hyperthyroidism	↑	↓	↑

Symbols: ↓, decreased, ↑, increased; ^a^ descending order of affinity. Abbreviations: DIO, deiodinase; DIO1, DIO type 1; DIO2, DIO type 2; DIO3, DIO type 3; K_m_, Michaelis–Menten constant, T2, 3,5-diiodothyronine; T3, 3,5,3’-triiodo-l-thyronine; T4, 3,3′,5,5′-tetraiodo-L-thyronine; rT3, 3,3′,5′-triio-dothyronine or reverse T3.

**Table 2:** Nonclinical studies addressing the TH-lung cancer association.

Ref.	Materials	Interventions/ Methods	Results
[93]	Murine 3LL tumor model.	SC injections (3 times/week) of T3 to induce hyperthyroid status with ↑T3 and ↓T4 levels.	➢ Significant inhibition of spontaneous pulmonary metastases of 3LL carcinoma and prolongation of survival of mice induced by hyperthyroid status with ↑T3 and ↓T4 levels.
		Treatment with T4 to induce hyperthyroid status with ↑T3 and ↑T4 levels.	➢ Increased primary tumor growth and development of pulmonary metastases of 3LL by ↑T3 and ↑T4 levels.
		MMI-induced hypothyroidism (↓T3 and ↓T4 levels).	➢ Suppressed primary and metastatic tumor growth, prolongation of survival by MMI-induced hypothyroidism.
			➢ Effect of T4, but not of T3, on immune cells.
[94]	Athymic nude mouse model of lung adenocarcinoma.	PTU-induced hypothyroidism or PTU plus LT4-induced hyperthyroid T4 levels.	➢ PTU-induced hypothyroidism led to tumor growth attenuation, compared to hyperthyroid T4 levels and control euthyroid animals.
		Inoculation of mice with lung adenocarcinoma cells.	➢ Tumor growth proceeded after euthyroidism restoration.
		Restoration of euthyroidism in hypothyroid mice via PTU withdrawal.	➢ No direct inhibitory effect of PTU on tumor growth.
[95]	18 SCLC and 29 NSCLC cell lines.	RT-PCR, direct sequencing, or methylation-specific PCR for TRβ1. Treatment with 5-aza-2-deoxy-cytidine and/or trichostatin-A in four cell lines.	➢ No TRβ1 expression in 61% of SCLCs and 48% of NSCLCs. ➢ TRβ1 promoter methylation in 67% of SCLCs and 45% of NSCLCs. ➢ No somatic TRβ1 mutation in NSCLC cell lines. ➢ Significant correlation of TRβ1 methylation status with loss of TRβ1 expression. ➢ Inhibition of DNA methylation resulted in restoration of TRβ1 expression.
[96]	Human NCI-H522 NSCLC cells and NCI-H510A SCLC cells.	Administration of Tetrac and ICI 182,780 (ICI), an ER antagonist (0.05–5 nM).	➢ Significant increase of PCNA abundance in both NCI-H522 cells and NCI-H510A cells by T4 at physiologic concentration (10^−7^ M) and T3 at supraphysiologic concentration. ➢ Activated ERα mediated the T4-induced, but not the T3-induced, cell proliferation initiated at integrin αvβ3.
[97]	Cultured human NSCLC H1299 cells in vitro.	Inhibition of TH–integrin αvβ3 interaction by unmodified tetrac, and by tetrac-NP.	➢ Tetrac and tetrac-NP attenuated tumor growth and tumor-related angiogenesis. ➢ Blockade of MAPK pathway inhibited TH-induced NSCLC cells proliferation.
	Tumor cell implants in the fertilized CAM system.	Pharmacologic inhibition of MAPK pathway.	
	Xenografts of H1299 cells in nude mouse.		
[98]	NSCLC (H522) cells.	Cell fractionation and separation of nucleoproteins.	TH induced:
		Immunoblotting and immunoprecipitation. Radioligand binding assay. In vitro phosphorylation. Chromatin immunoprecipitation. Quantitative real-time PCR.	➢ internalization and nuclear translocation of integrin αν monomer in human cancer cells ➢ phosphorylation of nuclear integrin αv and formation of complexes with nucleoproteins. Nuclear integrin αv acts as coactivator to promote gene expression in cancer cells.
[99]	Murine 3LL tumor model.	Administration of T4, methimazole, NO inhibitor L-NAME, T4 + L-NAME, methimazole + NAME, tetrac, T4 + tetrac, the iNOS inhibitor aminoguanidine (AG), and T4 + AG.	➢ T4 induced parallel increases in TW and NO-mediated angiogenesis. ➢ Tetrac attenuated TW in normal and T4-treated mice, but not angiogenesis in T4-treated mice.
		Untreated mice used as controls.	➢ NO blockade decreased TW, irrespectively of TH.
		Duration of all treatments: 6 weeks except for tetrac (the last 11 days).	➢ Inhibition of TR at αvß3 integrin decreased TW via, at least partially, enhanced AP activity.
		Evaluation of TW, Hb content, an index of tumor vascularization, VEGF, and AP activity 9 d after SC inoculation of mice with 3LL cells.	
[100]	Murine 3LL tumor model.	Treatment with T_3_ or T_4_ with or without tetrac.	➢ T4, but not T3, stimulated lung cancer growth via integrin ανβ3-mediated increase of neoangiogenesis.
		Serial in vivo imaging of bioluminescence to evaluate tumor progression.	➢ No direct effect of T4 or T3 on 3LL cells proliferation.
		TW record at the end of the experiment.	
		
		Neoangiogenesis assessment by IHC for CD31.	
		

Symbols: ↑, increased; ↓decreased. Abbreviations: AP, activator protein; CAM, chick chorioallantoic membrane; d, days; ERα, estrogen receptor α; Hb, hemoglobin; IHC, immunohistochemistry; 3LL, Lewis lung carcinoma; LT4, levothyroxine; MAPK, mitogen-activated protein kinase; MMI, methimazole; NSCLC, non-small-cell lung cancer; PCNA, proliferating cell nuclear antigen; PCR, polymerase chain reaction; PTU, Propylthiouracil; RT-PCR, reverse transcription PCR; SC, sub-cutaneous; SCLC, small-cell lung cancer; TH, thyroid hormones; TW, tumor weight; TR, thyroid receptor; VEGF, vascular endothelial growth factor.

**Table 3:** Clinical studies addressing the thyroid hormones–lung cancer association.

Ref.	Type and Aim of Study	Results
[6]	Case report of a 69-years-old male patient with metastatic NSCLC treated with radiation and chemotherapy with amiodarone-induced myxedema coma.	➢ Spontaneous remission of NSCLC after myxedema coma during a 4-year FU.
[101]	Retrospective case control study of 1979 patients with NSCLC and SCLC (stages I–IV) to assess the incidence and the prognostic significance of primary hypothyroidism.	➢ Incidence of hypothyroidism: 4.2%. ➢ Female to male: 3.35:1. ➢ Median survival in hypothyroid group vs. euthyroid group: 14.5 mo vs. 11.1 mo; *p* = 0.014 (stages I–V) 11 mo vs. 5 mo; *p* = 0.0018 (stage IV) ➢ Primary hypothyroidism was significant prognostic factor for favorable clinical outcome of NSCLC and SCLC.
[102]	A research study of 9 NSCLC patients and 11 healthy individuals to assess the pattern of HPT axis function in a 24 h sleep-wake schedule.	➢ Circadian rhythm synchronized to the 24 h environmental light-dark cycle similar between NSCLC patients and healthy participants. ➢ NSCLC patients vs. healthy participants: lower 24 h means of TSH levels higher 24 h means of TRH, fT4, and IL-2 levels
[103]	Prospective study of 29,691 individuals without previously known thyroid disease to assess the association of thyroid function with cancer risk.	➢ Correlation of TSH <0.50 mU/L with higher risk for any cancer type compared to any other TSH levels. ➢ TSH <0.50 mU/L compared to TSH 0.50–1.4 mU/L: ↑risk for any cancer (adjusted HR, 1.34) ↑risk for lung cancer: HR adjusted for smoking: 2.34 unadjusted HR: 2.60 HR excluding the first 2 years of FU: 2.91 ➢ Subclinical hyperthyroidism compared to euthyroidism (TSH: 0.50–1.4 mU/L): ↑risk for lung cancer: HR, 2.55; 95% CI, 1.22–5.35. ➢ Stronger association of overt hyperthyroidism compared to subclinical hyperthyroidism with lung cancer risk.
[104]	Prospective, population-based, cohort study of 10,318 individuals to assess the association of thyroid function with cancer risk.	➢ Compared to fT4 of the lowest tertile (0.12–1.14 ng/dL), significant association of fT_4_ of the highest tertile (1.29–4.73 ng/dL) with: 1.79-fold ↑ risk of lung cancer 1.13-fold ↑ risk of any solid cancer 1.14-fold ↑ risk of breast cancer ➢ No association between TSH levels and lung cancer risk.
[105]	Prospective cohort study of a community-dwelling population of 3649 participants aged 25–84 years in Western Australia to assess the association of thyroid function with cancer risk.	➢ No association of TSH, fT4 or anti-TPOAbs with lung cancer.
[106]	Research study of correlation of sales of LT_4_ in 2009 with the prevalence of lung cancer (as well as breast, colorectal, gastric and cancer) in women of 30–84 years-old in 18 Italian regions treated with LT4 during 2010.	➢ Significant correlation of LT4 administration with development of lung cancer corrected for smoking and age, but not with any other cancer types.
[95]	Research study of analysis of TRβ1 methylation in 116 NSCLC surgical specimens.	➢ TRβ1 methylation detected in 47% of NSCLC surgical specimens. ➢ No significant association of methylation with any clinico-pathological parameters or mutations of KRAS and EGFR.

Symbols: ↑, increased. Abbreviations: CI, confidence interval; EGFR, epidermal growth factor receptor; fT4, free thyroxine; FU; follow-up; h, hours; HR, hazard ratio; LT_4_, levothyroxine; mo; months; NSCLC, non-small cell lung cancer; Ref., reference; SCLC, small cell lung cancer; anti-TPOAbs, anti-thyroid peroxidase antibodies; TRβ1, thyroid hormone receptor beta 1; TRH, thyrotropin-releasing hormone; TSH, thyroid-stimulating hormone; vs, versus.

**Table 4:** Representative studies addressing the thyroid disorders related to ICPi and TKI in lung cancer patients.

Ref	Patients/Methods	Results
[113]	Prospective study of 51 patients with advanced NSCLC treated with pembrolizumab in the setting of KEYNOTE-001 (Clinical Trial Registry ID: NCT01295827).	➢ Incidence of thyroid disorders: 21%. ➢ Strong association of thyroid disorders with anti-thyroid antibodies. ➢ Median onset time of hypothyroidism: 98 d (20–231 d). ➢ Hypothyroidism was preceded by transient hyperthyroidism in 6 of 10 patients. ➢ Patients without thyroid disorders vs. patients with thyroid disorders: median OS: 40 mo vs. 14 mo; HR, 0.29; 95% CI, 0.09–0.94; *p =* 0.029) median PFS: 8 mo vs. 2 mo; HR, 0.58; 95% CI 0.27–1.21; *p* = 0.14
[114]	Prospective study of 134 patients with histologically confirmed stage IIIB/IV NSCLC treated with nivolumab due to disease progression after one or two lines of treatment.	➢ Incidence of ir thyroid disorders: 29.9%. ➢ Patients with thyroid disorders vs. no ir thyroid disorders: longer OS (29.8 mo vs. 8.1 mo; (*p* < 0.001) longer PFS (8.7 months vs. 1.7 months; *p* < 0.001) ➢ Ir thyroid disorders: independent predictive factor of OS and PFS. ➢ Ir thyroid disorders: independent predictive factor of survival, irrespectively of their severity and subtype.
[115]	Retrospective analysis of 111 NSCLC patients treated with nivolumab.	➢ Significant association of low fT4 levels compared to no low fT4 levels with: longer PFS (not reached versus 67 days; HR, 0.297; *p* = 0.010) longer median OS (not reached versus 556 days, HR, 0.139; *p* = 0.020) ➢ Significant association of low fT4 levels with the T allele of rs 1411262 (*p* = 0.0073) and the A allele of rs 822,339 (*p* = 0.0204).
[116]	Retrospective analysis of 126 patients with advanced solid tumors (NSCLC, renal cell carcinoma, metastatic melanoma) treated with PD-1 inhibitors (nivolumab, pembrolizumab).	➢ Incidence of ir thyroid dysfunction: 23% Hypothyroidism: 15.1% (subclinical: 11.9%, overt: 3.2%) Hyperthyroidism: 8.0% (subclinical: 4.8%, overt: 3.2%) ➢ Median onset time: 8.7 ± 6.8 weeks (10.4 ± 7.6 weeks for hypothyroidism, 5.4 ± 3.0 weeks for hyperthyroidism). 63.2% of hypothyroid patients had previously been treated with TKI. ➢ Pretreatment with TKI: major predisposing factor for ir hypothyroidism (OR 9.2, 95% CI: 1.4–59.9, *p* = 0.020).
[118]	Systematic review of 24 eligible trials enrolling 6,678 patients treated with sunitinib. A meta-analysis of seven randomized trials enrolling 2,787 subjects.	➢ Incidence of all-and high-grade of sunitinib-related hypothyroidism: 9.8% (95% CI 7.3–12.4%) and 0.4% (95% CI 0.3–0.5%), respectively. ➢ RR of hypothyroidism: 13.95 (95% CI, 6.91–28.15; *p* < 0.00001, for all grade hypothyroidism), and 4.78 (95% CI, 1.09–20.84; *p* = 0.04, for high-grade hypothyroidism). ➢ Significantly higher incidence of all-grade hypothyroidism (*p* = 0.02) related to duration of treatment with sunitinib longer than mean treatment duration of all trials (3.5 months).
[120]	Prospective observational study of 50 NSCLC patients treated with EGFR and ALK inhibitors over a period of 15 mo.	➢ Prevalence of thyroid dysfunction: 8% subclinical: 4% overt thyroid dysfunction: 4% ➢ Two EGFR inhibitors (erlotinib, gefitinib) and two ALK inhibitors (ceritinib, crizotinib) were related to thyroid dysfunction, with onset time of 1 month after TKI initiation. ➢ All patients were asymptomatic. ➢ Overt thyroid dysfunction: hypothyroidism (all cases) ➢ Subclinical thyroid dysfunction: hypothyroidism (half of cases) hyperthyroidism (the other half of cases) ➢ Treatment with TKI yielded clinical benefit for all patients with thyroid dysfunction. ➢ No case of TKI interruption or withdrawal.
[121]	Retrospective review of 197 patients with various cancer types including NSCLC from 4 clinical trials that included therapy with at least one TKI agent.	➢ Incidence of TKI-induced hypothyroidism: 26%. ➢ Higher clinical benefit rates in patients with new-onset hypothyroidism (50%) compared to patients without hypothyroid-ism (34%). ➢ In the univariate model, OR for new-onset hypothyroidism: 1.9 [95% CI, 1.0, 3.6; *p* = 0.05].
[122]	Phase III ALTER-0303 trial (Clinical Trial Registry ID: NCT 02388919).	➢ Incidence of hypothyroidism related to TKI targeting VEGFR, FGFR, PDGF-R, and c-Kit in anlotinib group vs. placebo group: ➢ 46.6% (all grades), 0.3 (grade 3–4) vs. 8.4% (all grades), 0% (grade 3–4).

Abbreviations: ALK, anaplastic lymphoma kinase; CI, confidence interval; c-Kit, and stem cell factor receptor, d, days; EGFR, epidermal growth factor receptor; FGFR, fibroblast growth factor receptor, HR, hazard ratio; mo, months; ICPi, immune checkpoint inhibitors; NSCLC, non-small cell lung cancer; OR, odds ratio; OS, overall survival; PD-1; programmed cell death protein 1; PDGF-R, platelet-derived growth factor receptor; PFS, progression free survival; Ref, reference; RR, relative risk; TKI, tyrosine kinase inhibitors, VEGFR, vascular endothelial growth factor receptor.

## References

[1] Khatawkar A.V., Awati S.M. (2015). Thyroid gland-Historical aspects, Embryology, Anatomy and Physiology. IAIM.

[2] Salvatore D., Davies T., Sohlumberger M., Hay I., Larsen P., Melmed S., Koenig R., Rosen C., Auchus R., Goldfine A. (2011). Chapter 11-Thyroid Physiology and Diagnostic Evaluation of Patients with Thyroid Disorders. Williams Textbook of Endocrinology.

[3] Davis P.J., Leonard J.L., Lin H.-Y., Leinung M., Mousa S.A. (2018). Molecular Basis of Nongenomic Actions of Thyroid Hormone. Vitam. Horm..

[4] Krashin E., Piekiełko-Witkowska A., Ellis M., Ashur-Fabian O. (2019). Thyroid Hormones and Cancer: A Comprehensive Review of Preclinical and Clinical Studies. Front. Endocrinol..

[5] Sap J., Muñoz A., Damm K., Goldberg Y., Ghysdael J., Leutz A., Beug H., Vennström B. (1986). The c-erb-A protein is a high-affinity receptor for thyroid hormone. Nature.

[6] Hercbergs A., Leith J.T. (1993). Spontaneous Remission of Metastatic Lung Cancer Following Myxedema Coma--An Apoptosis-Related Phenomenon?. J. Natl. Cancer Inst..

[7] Gauthier B.R., Sola-García A., Cáliz-Molina M.Á., Lorenzo P.I., Cobo-Vuilleumier N., Capilla-González V., Martin-Montalvo A. (2020). Thyroid hormones in diabetes, cancer, and aging. Aging Cell.

[8] Liu Y.-C., Yeh C.-T., Lin K.-H. (2019). Molecular Functions of Thyroid Hormone Signaling in Regulation of Cancer Progression and Anti-Apoptosis. Int. J. Mol. Sci..

[9] Sung H., Ferlay J., Siegel R.L., Laversanne M., Soerjomataram I., Jemal A., Bray F. (2021). Global Cancer Statistics 2020: GLOBOCAN Estimates of Incidence and Mortality Worldwide for 36 Cancers in 185 Countries. CA Cancer J. Clin..

[10] https://seer.cancer.gov/statfacts/html/lungb.html.

[11] https://www.cancer.org/cancer/lung-cancer/about/what-is.html.

[12] Yuan M., Huang L.-L., Chen J.-H., Wu J., Xu Q. (2019). The emerging treatment landscape of targeted therapy in non-small-cell lung cancer. Signal Transduct. Target. Ther..

[13] Hirsch F.R., Scagliotti G.V., Mulshine J.L., Kwon R., Curran W.J., Wu Y.-L., Paz-Ares L. (2017). Lung cancer: Current therapies and new targeted treatments. Lancet.

[14] Howlader N., Forjaz G., Mooradian M.J., Meza R., Kong C.Y., Cronin K.A., Mariotto A.B., Lowy D.R., Feuer E.J. (2020). The Effect of Advances in Lung-Cancer Treatment on Population Mortality. N. Engl. J. Med..

[15] Farago A.F., Azzoli C.G. (2017). Beyond ALK and ROS1: RET, NTRK, EGFR and BRAF gene rearrangements in non-small cell lung cancer. Transl. Lung Cancer Res..

[16] Wang F., Han S., Yang J., Yan W., Hu G. (2021). Knowledge-Guided “Community Network” Analysis Reveals the Functional Modules and Candidate Targets in Non-Small-Cell Lung Cancer. Cells.

[17] Brahmer J., Reckamp K.L., Baas P., Crinò L., Eberhardt W.E.E., Poddubskaya E., Antonia S., Pluzanski A., Vokes E.E., Holgado E. (2015). Nivolumab versus Docetaxel in Advanced Squamous-Cell Non–Small-Cell Lung Cancer. N. Engl. J. Med..

[18] Kazandjian D., Khozin S., Blumenthal G., Zhang L., Tang S., Libeg M., Kluetz P., Sridhara R., Keegan P., Pazdur R. (2016). Benefit-Risk Summary of Nivolumab for Patients with Metastatic Squamous Cell Lung Cancer After Platinum-Based Chemotherapy: A Report from the US Food and Drug Administration. JAMA Oncol..

[19] Kazandjian D., Suzman D.L., Blumenthal G., Mushti S., He K., Libeg M., Keegan P., Pazdur R. (2016). FDA Approval Summary: Nivolumab for the Treatment of Metastatic Non-Small Cell Lung Cancer with Progression on or After Platinum-Based Chemotherapy. Oncologist.

[20] Lim S.M., Hong M.H., Kim H.R. (2020). Immunotherapy for Non-small Cell Lung Cancer: Current Landscape and Future Perspectives. Immune Netw..

[21] Pakkala S., Ramalingam S.S. (2018). Personalized therapy for lung cancer: Striking a moving target. JCI Insight.

[22] Ding P., Ouyang W., Luo J., Kwoh C.-K. (2020). Heterogeneous information network and its application to human health and disease. Brief. Bioinform..

[23] Montesinos M.D.M., Pellizas C.G. (2019). Thyroid Hormone Action on Innate Immunity. Front. Endocrinol..

[24] Cooper D.S., Landenson P.W., Gardner D., Shoback D. (2011). Chapter 7. The Thyroid Gland. Greenspan’s Basic and Clinical Endocrinology.

[25] Saponaro F., Sestito S., Runfola M., Rapposelli S., Chiellini G. (2020). Selective Thyroid Hormone Receptor-Beta (TRβ) Agonists: New Perspectives for the Treatment of Metabolic and Neurodegenerative Disorders. Front. Med..

[26] Tancevski I., Rudling M., Eller P. (2011). Thyromimetics: A journey from bench to bed-side. Pharmacol. Ther..

[27] Moreno M., de Lange P., Lombardi A., Silvestri E., Lanni A., Goglia F. (2008). Metabolic Effects of Thyroid Hormone Derivatives. Thyroid.

[28] Lin H.-Y., Tang H.-Y., Leinung M., Mousa S.A., Hercbergs A., Davis P.J. (2019). Action of Reverse T3 on Cancer Cells. Endocr. Res..

[29] Laurino A., Landucci E., Raimondi L. (2018). Central Effects of 3-Iodothyronamine Reveal a Novel Role for Mitochondrial Monoamine Oxidases. Front. Endocrinol..

[30] Louzada R.A., Carvalho D.P. (2018). Similarities and Differences in the Peripheral Actions of Thyroid Hormones and Their Metabolites. Front. Endocrinol..

[31] Senese R., de Lange P., Petito G., Moreno M., Goglia F., Lanni A. (2018). 3,5-Diiodothyronine: A Novel Thyroid Hormone Metabolite and Potent Modulator of Energy Metabolism. Front. Endocrinol..

[32] Giammanco M., di Liegro C.M., Schiera G., di Liegro I. (2020). Genomic and Non-Genomic Mechanisms of Action of Thyroid Hormones and Their Catabolite 3,5-Diiodo-L-Thyronine in Mammals. Int. J. Mol. Sci..

[33] Ortiga-Carvalho T.M., Chiamolera M.I., Pazos-Moura C.C., Wondisford F.E. (2016). Hypothalamus-pituitary-thyroid axis. Compr. Physiol..

[34] Mendoza A., Hollenberg A.N. (2017). New insights into thyroid hormone action. Pharmacol. Ther..

[35] Keestra S., Tabor V.H., Alvergne A. (2021). Reinterpreting patterns of variation in human thyroid function: An evolu-tionary ecology perspective. Evol. Med. Public Health.

[36] Davis F.B., Cody V., Davis P.J., Borzynski L.J., Blas S.D. (1983). Stimulation by thyroid hormone analogues of red blood cell Ca^2+^-ATPase activity in vitro. Correlations between hormone structure and biological activity in a human cell system. J. Biol. Chem..

[37] Segal J., Ingbar S.H. (1989). Evidence that an Increase in Cytoplasmic Calcium Is the Initiating Event in Certain Plasma Membrane-Mediated Responses to 3,5,3′-Triiodothyronine in Rat Thymocytes. Endocrinology.

[38] Segal J., Ingbar S.H. (1990). 3,5,3′-Tri-iodothyronine enhances sugar transport in rat thymocytes by increasing the intrinsic activity of the plasma membrane sugar transporter. J. Endocrinol..

[39] Siegrist-Kaiser C.A., Juge-Aubry C., Tranter M.P., Ekenbarger D.M., Leonard J.L. (1990). Thyroxine-dependent modulation of actin polymerization in cultured astrocytes. A novel, extranuclear action of thyroid hormone. J. Biol. Chem..

[40] Sterling K., Brenner M.A., Sakurada T. (1980). Rapid Effect of Triiodothyronine on the Mitochondrial Pathway in Rat Liver In Vivo. Science.

[41] Flamant F., Cheng S.-Y., Hollenberg A.N., Moeller L.C., Samarut J., Wondisford F.E., Yen P.M., Refetoff S. (2017). Thyroid Hormone Signaling Pathways: Time for a More Precise Nomenclature. Endocrinology.

[42] Weikum E.R., Liu X., Ortlund E.A. (2018). The nuclear receptor superfamily: A structural perspective. Protein Sci..

[43] Cheng S.-Y., Leonard J.L., Davis P.J. (2010). Molecular Aspects of Thyroid Hormone Actions. Endocr. Rev..

[44] de Luca R., Davis P.J., Lin H.-Y., Gionfra F., Percario Z.A., Affabris E., Pedersen J.Z., Marchese C., Trivedi P., Anastasiadou E. (2021). Thyroid Hormones Interaction with Immune Response, Inflammation and Non-thyroidal Illness Syndrome. Front. Cell Dev. Biol..

[45] Zhang J., Roggero V.R., Allison L.A. (2018). Nuclear Import and Export of the Thyroid Hormone Receptor. Vitam. Horm..

[46] Nakano K., Matsushita A., Sasaki S., Misawa H., Nishiyama K., Kashiwabara Y., Nakamura H. (2004). Thyroid-hormone-dependent negative regulation of thyrotropin beta gene by thyroid hormone receptors: Study with a new experimental system using CV1 cells. Biochem. J..

[47] Weitzel J.M. (2008). To bind or not to bind—How to down-regulate target genes by liganded thyroid hormone receptor?. Thyroid. Res..

[48] Kashiwabara Y., Sasaki S., Matsushita A., Nagayama K., Ohba K., Iwaki H., Matsunaga H., Suzuki S., Misawa H., Ishizuka K. (2009). Functions of PIT1 in GATA2-dependent transactivation of the thyrotropin β promoter. J. Mol. EnDocrinol..

[49] Fonseca T.L., Correa-Medina M., Campos M.P.O., Wittmann G., Werneck-de-Castro J.P., e Drigo R.A., Mora-Garzon M., Ueta C.B., Caicedo A., Fekete C. (2013). Coordination of hypothalamic and pituitary T3 production regulates TSH expression. J. Clin. Investig..

[50] Matsunaga H., Sasaki S., Suzuki S., Matsushita A., Nakamura H., Nakamura H.M., Hirahara N., Kuroda G., Iwaki H., Ohba K. (2015). Essential Role of GATA2 in the Negative Regulation of Type 2 Deiodinase Gene by Liganded Thyroid Hormone Receptor β2 in Thyrotroph. PLoS ONE.

[51] Davis F.B., Mousa S.A., O’Connor L., Mohamed S., Lin H.-Y., Cao H.J., Davis P.J. (2004). Proangiogenic Action of Thyroid Hormone Is Fibroblast Growth Factor–Dependent and Is Initiated at the Cell Surface. Circ. Res..

[52] Bergh J.J., Lin H.Y., Lansing L., Mohamed S.N., Davis F.B., Mousa S., Davis P.J. (2005). Integrin alphaVbeta3 contains a cell surface receptor site for thyroid hormone that is linked to activation of mitogen-activated protein kinase and induction of angiogenesis. Endocrinology.

[53] Havaki S., Kouloukoussa M., Amawi K., Drosos Y., Arvanitis L.D., Goutas N., Vlachodimitropoulos D., Vassilaros S.D., Katsantoni E.Z., Voloudakis-Baltatzis I. (2007). Altered expression pattern of integrin alphavbeta3 correlates with actin cytoskeleton in primary cultures of human breast cancer. Cancer Cell Int..

[54] Saltel F., Chabadel A., Bonnelye E., Jurdic P. (2008). Actin cytoskeletal organisation in osteoclasts: A model to decipher transmigration and matrix degradation. Eur. J. Cell Biol..

[55] Koutsioumpa M., Polytarchou C., Courty J., Zhang Y., Kieffer N., Mikelis C., Skandalis S.S., Hellman U., Iliopoulos D., Papadimitriou E. (2013). Interplay between αvβ3 Integrin and Nucleolin Regulates Human Endothelial and Glioma Cell Migration. J. Biol. Chem..

[56] Colin D., Limagne E., Jeanningros S., Jacquel A., Lizard G., Athias A., Gambert P., Hichami A., Latruffe N., Solary E. (2011). Endocytosis of Resveratrol via Lipid Rafts and Activation of Downstream Signaling Pathways in Cancer Cells. Cancer Prev. Res..

[57] Reuning U. (2011). Integrin αvβ3 promotes vitronectin gene expression in human ovarian cancer cells by implicating rel transcription factors. J. Cell. Biochem..

[58] Umemoto T., Yamato M., Ishihara J., Shiratsuchi Y., Utsumi M., Morita Y., Tsukui H., Terasawa M., Shibata T., Nishida K. (2012). Integrin-αvβ3 regulates thrombopoietin-mediated maintenance of hematopoietic stem cells. Blood.

[59] Roth P., Silginer M., Goodman S.L., Hasenbach K., Thies S., Maurer G., Schraml P., Tabatabai G., Moch H., Tritschler I. (2013). Integrin control of the transforming growth factor-β pathway in glioblastoma. Brain.

[60] Cody V., Davis P.J., Davis F.B. (2007). Molecular modeling of the thyroid hormone interactions with alpha v beta 3 integrin. Steroids.

[61] Ho Y., Li Z.-L., Shih Y.-J., Chen Y.-R., Wang K., Whang-Peng J., Lin H.-Y., Davis P.J. (2020). Integrin αvβ3 in the Mediating Effects of Dihydrotestosterone and Resveratrol on Breast Cancer Cell Proliferation. Int. J. Mol. Sci..

[62] Davis P.J., Davis F.B., Mousa S.A., Luidens M.K., Lin H.Y. (2011). Membrane receptor for thyroid hormone: Physiologic and phar-macologic implications. Annu. Rev. Pharmacol. Toxicol..

[63] Hercbergs A. (2019). Clinical Implications and Impact of Discovery of the Thyroid Hormone Receptor on Integrin αvβ3—A Review. Front. Endocrinol..

[64] Davis P.J., Mousa S.A., Schechter G.P., Lin H.-Y. (2020). Platelet ATP, Thyroid Hormone Receptor on Integrin αvβ3 and Cancer Metastasis. Horm. Cancer.

[65] Davis P.J., Leonard J.L., Davis F.B. (2008). Mechanisms of nongenomic actions of thyroid hormone. Front. Neuroendocr..

[66] Davis P.J., Glinsky G.V., Lin H.-Y., Leith J.T., Hercbergs A., Tang H.-Y., Ashur-Fabian O., Incerpi S., Mousa S.A. (2015). Cancer Cell Gene Expression Modulated from Plasma Membrane Integrin αvβ3 by Thyroid Hormone and Nanoparticulate Tetrac. Front. Endocrinol..

[67] Davis P.J., Mousa S.A., Lin H.-Y. (2021). Nongenomic Actions of Thyroid Hormone: The Integrin Component. Physiol. Rev..

[68] Huang T.-Y., Chang T.-C., Chin Y.-T., Pan Y.-S., Chang W.-J., Liu F.-C., Hastuti E.D., Chiu S.-J., Wang S.-H., Changou C.A. (2020). NDAT Targets PI3K-Mediated PD-L1 Upregulation to Reduce Proliferation in Gefitinib-Resistant Colorectal Cancer. Cells.

[69] Davis P.J., Goglia F., Leonard J. (2016). L Nongenomic actions of thyroid hormone. Nat. Rev. Endocrinol..

[70] Ashur-Fabian O., Zloto O., Fabian I., Tsarfaty G., Ellis M., Steinberg D.M., Hercbergs A., Davis P.J., Fabian I.D. (2019). Tetrac Delayed the Onset of Ocular Melanoma in an Orthotopic Mouse Model. Front. Endocrinol..

[71] Davis P.J., Incerpi S., Lin H.-Y., Tang H.-Y., Sudha T., Mousa S.A. (2015). Thyroid Hormone and P-Glycoprotein in Tumor Cells. BioMed Res. Int..

[72] Hallen A., Cooper A.J.L., Jamie J.F., Karuso P. (2015). Insights into Enzyme Catalysis and Thyroid Hormone Regulation of Cerebral Ketimine Reductase/μ-Crystallin Under Physiological Conditions. Neurochem. Res..

[73] Goemann I.M., Romitti M., Meyer E.L.S., Wajner S.M., Maia A.L. (2017). Role of thyroid hormones in the neoplastic process: An overview. Endocr. Relat. Cancer.

[74] Hanahan D., Weinberg R.A. (2000). The hallmarks of cancer. Cell.

[75] Hanahan D., Weinberg R.A. (2011). Hallmarks of Cancer: The Next Generation. Cell.

[76] Chen C.-Y., Chung I.-H., Tsai M.-M., Tseng Y.-H., Chi H.-C., Tsai C.-Y., Lin Y.-H., Wang Y.-C., Chen C.-P., Wu T.-I. (2014). Thyroid hormone enhanced human hepatoma cell motility involves brain-specific serine protease 4 activation via ERK signaling. Mol. Cancer.

[77] Lin Y.-H., Liao C.-J., Huang Y.-H., Wu M.-H., Chi H.-C., Wu S.-M., Chen C.-Y., Tseng Y.-H., Tsai C.-Y., Chung I.-H. (2013). Thyroid hormone receptor represses miR-17 expression to enhance tumor metastasis in human hepatoma cells. Oncogene.

[78] Lin Y.-H., Wu M.-H., Liao C.-J., Huang Y.-H., Chi H.-C., Wu S.-M., Chen C.-Y., Tseng Y.-H., Tsai C.-Y., Chung I.-H. (2015). Repression of microRNA-130b by thyroid hormone enhances cell motility. J. Hepatol..

[79] Huang S.A., Tu H.M., Harney J.W., Venihaki M., Butte A.J., Kozakewich H.P.W., Fishman S.J., Larsen P.R. (2000). Severe Hypothyroidism Caused by Type 3 Iodothyronine Deiodinase in Infantile Hemangiomas. N. Engl. J. Med..

[80] Liao C.-H., Yeh S.-C., Huang Y.-H., Chen R.-N., Tsai M.-M., Chen W.-J., Chi H.-C., Tai P.-J., Liao C.-J., Wu S.-M. (2010). Positive regulation of spondin 2 by thyroid hormone is associated with cell migration and invasion. Endocr. Relat. Cancer.

[81] Chi H.-C., Liao C.-H., Huang Y.-H., Wu S.-M., Tsai C.-Y., Liao C.-J., Tseng Y.-H., Lin Y.-H., Chen C.-Y., Chung I.-H. (2013). Thyroid hormone receptor inhibits hepatoma cell migration through transcriptional activation of Dickkopf 4. Biochem. Biophys. Res. Commun..

[82] Liao C.-H., Yeh C.-T., Huang Y.-H., Wu S.-M., Chi H.-C., Tsai M.-M., Tsai C.-Y., Liao C.-J., Tseng Y.-H., Lin Y.-H. (2012). Dickkopf 4 positively regulated by the thyroid hormone receptor suppresses cell invasion in human hepatoma cells. Hepatolology.

[83] Suhane S., Ramanujan V.K. (2011). Thyroid hormone differentially modulates Warburg phenotype in breast cancer cells. Biochem. Biophys. Res. Commun..

[84] Kowalik M.A., Puliga E., Cabras L., Sulas P., Petrelli A., Perra A., Ledda-Columbano G.M., Morandi A., Merlin S., Orrù C. (2020). Thyroid hormone inhibits hepatocellular carcinoma progression via induction of differentiation and metabolic reprogramming. J. Hepatol..

[85] Lin H.-Y., Chin Y.-T., Nana A.W., Shih Y.-J., Lai H.-Y., Tang H.-Y., Leinung M., Mousa S.A., Davis P.J. (2016). Actions of l-thyroxine and Nano-diamino-tetrac (Nanotetrac) on PD-L1 in cancer cells. Steroids.

[86] Perrotta C., Buldorini M., Assi E., Cazzato D., de Palma C., Clementi E., Cervia D. (2014). The thyroid hormone triiodothyronine controls macrophage maturation and functions: Protective role during inflammation. Am. J. Pathol..

[87] Szabó J., Fóris G., Mezösi E., Nagy E.V., Paragh G., Sztojka I., Leövey A. (1996). Parameters of respiratory burst and arachidonic acid metabolism in polymorphonuclear granulocytes from patients with various thyroid diseases. Exp. Clin. Endocrinol. Diabetes.

[88] Provinciali M., Fabris N. (1990). Modulation of lymphoid cell sensitivity to interferon by thyroid hormones. J. Endocrinol. Investig..

[89] Lee E.K., Sunwoo J.B. (2019). Natural Killer Cells and Thyroid Diseases. Endocrinol. Metab..

[90] Schmohl K.A., Müller A.M., Wechselberger A., Rühland S., Salb N., Schwenk N., Heuer H., Carlsen J., Göke B., Nelson P.J. (2015). Thyroid hormones and tetrac: New regulators of tumour stroma formation via integrin αvβ3. Endocr. Relat. Cancer.

[91] Zambrano A., García-Carpizo V., Gallardo M.E., Villamuera R., Gómez-Ferrería M.A., Pascual A., Buisine N., Sachs L.M., Garesse R., Aranda A. (2014). The thyroid hormone receptor β induces DNA damage and premature senescence. J. Cell Biol..

[92] Hoare M., Narita M. (2018). The Power behind the Throne: Senescence and the Hallmarks of Cancer. Annu. Rev. Cancer Biol..

[93] Kinoshita S., Sone S., Yamashita T., Tsubura E., Ogura T. (1991). Effects of experimental hyper- and hypothyroidism on natural defense activities against Lewis lung carcinoma and its spontaneous pulmonary metastases in C57BL/6 mice. Tokushima J. Exp. Med..

[94] Theodossiou C., Skrepnik N., Robert E.G., Prasad C., Axelrad T.W., Schapira D.V., Hunt J.D. (1999). Propylthiouracil-induced hypothyroidism reduces xenograft tumor growth in athymic nude mice. Cancer.

[95] Iwasaki Y., Sunaga N., Tomizawa Y., Imai H., Iijima H., Yanagitani N., Horiguchi K., Yamada M., Mori M. (2010). Epigenetic inactivation of the thyroid hormone receptor beta1 gene at 3p24.2 in lung cancer. Ann. Surg. Oncol..

[96] Meng R., Tang H.-Y., Westfall J., London D., Cao J.H., Mousa S.A., Luidens M., Hercbergs A., Davis F.B., Davis P.J. (2011). Crosstalk between Integrin αvβ3 and Estrogen Receptor-α Is Involved in Thyroid Hormone-Induced Proliferation in Human Lung Carcinoma Cells. PLoS ONE.

[97] Mousa S.A., Yalcin M., Bharali D.J., Meng R., Tang H.-Y., Lin H.-Y., Davis F.B., Davis P.J. (2012). Tetraiodothyroacetic acid and its nanoformulation inhibit thyroid hormone stimulation of non-small cell lung cancer cells in vitro and its growth in xenografts. Lung Cancer.

[98] Lin H.-Y., Su Y.-F., Hsieh M.-T., Lin S., Meng R., London D., Lin C., Tang H.-Y., Hwang J., Davis F.B. (2013). Nuclear monomeric integrin αv in cancer cells is a coactivator regulated by thyroid hormone. FASEB J..

[99] Carmona-Cortés J., Rodríguez-Gómez I., Wangensteen R., Banegas I., García-Lora Á.M., Quesada A., Osuna A., Vargas F. (2014). Effect of thyroid hormone–nitric oxide interaction on tumor growth, angiogenesis, and aminopeptidase activity in mice. Tumour Biol..

[100] Latteyer S., Christoph S., Theurer S., Hönes G.S., Schmid K.W., Führer D., Moeller L.C. (2019). Thyroxine promotes lung cancer growth in an orthotopic mouse model. Endocr. Relat. Cancer.

[101] Hercbergs A., Mason J., Reddy C., Elson P. (2004). Thyroid hormones and lung cancer: Primary hypothyroidism is prognostically significant for survival in lung cancer. Cancer Res..

[102] Mazzoccoli G., Pazienza V., Piepoli A., Muscarella L.A., Giuliani F., Sothern R.B. (2012). Alteration of Hypothalamic–Pituitary–Thyroid Axis Function in Non-Small-Cell Lung Cancer Patients. Integr. Cancer Ther..

[103] Hellevik A.I., Åsvold B.O., Bjøro T., Romundstad P., Nilsen T.I.L., Vatten L.J. (2009). Thyroid Function and Cancer Risk: A Prospective Population Study. Cancer Epidemiol. Biomark. Prev..

[104] Khan S.R., Chaker L., Ruiter R., Aerts J.G.J.V., Hofman A., Dehghan A., Franco O.H., Stricker B.H.C., Peeters R.P. (2016). Thyroid Function and Cancer Risk: The Rotterdam Study. J. Clin. Endocrinol. Metab..

[105] Chan Y.X., Knuiman M.W., Divitini M.L., Brown S.J., Walsh J., Yeap B.B. (2017). Lower TSH and higher free thyroxine predict incidence of prostate but not breast, colorectal or lung cancer. Eur. J. Endocrinol..

[106] Cornelli U., Belcaro G., Recchia M., Finco A. (2013). Levothyroxine and lung cancer in females: The importance of oxidative stress. Reprod. Biol. Endocrinol..

[107] Bhattacharya S., Goyal A., Kaur P., Singh R., Kalra S. (2020). Anticancer Drug-induced Thyroid Dysfunction. Eur. Endocrinol..

[108] El Sabbagh R., Azar N.S., Eid A.A., Azar S.T. (2020). Thyroid Dysfunctions Due to Immune Checkpoint Inhibitors: A Review. Int. J. Gen. Med..

[109] Lee H., Hodi F.S., Giobbie-Hurder A., Ott P.A., Buchbinder E.I., Haq R., Tolaney S., Barroso-Sousa R., Zhang K., Donahue H. (2017). Characterization of Thyroid Disorders in Patients Receiving Immune Checkpoint Inhibition Therapy. Cancer Immunol. Res..

[110] Illouz F., Drui D., Caron P., Cao C.D. (2018). Expert opinion on thyroid complications in immunotherapy. Ann. D’endocrinologie.

[111] Chalan P., di Dalmazi G., Pani F., de Remigis A., Corsello A., Caturegli P. (2018). Thyroid dysfunctions secondary to cancer immunotherapy. J. Endocrinol. Investig..

[112] Kurimoto C., Inaba H., Ariyasu H., Iwakura H., Ueda Y., Uraki S., Takeshima K., Furukawa Y., Morita S., Yamamoto Y. (2020). Predictive and sensitive biomarkers for thyroid dysfunctions during treatment with immune-checkpoint inhibitors. Cancer Sci..

[113] Osorio J.C., Ni A., Chaft J.E., Pollina R., Kasler M.K., Stephens D., Rodriguez C., Cambridge L., Rizvi H., Wolchok J.D. (2017). Antibody-mediated thyroid dysfunction during T-cell checkpoint blockade in patients with non-small-cell lung cancer. Ann. Oncol..

[114] Thuillier P., Joly C., Alavi Z., Crouzeix G., Descourt R., Quere G., Kerlan V., Roudaut N. (2021). Thyroid dysfunction induced by immune checkpoint inhibitors is associated with a better progression-free survival and overall survival in non-small cell lung cancer: An original cohort study. Cancer Immunol. Immunother..

[115] Funazo T.Y., Nomizo T., Ozasa H., Tsuji T., Yasuda Y., Yoshida H., Sakamori Y., Nagai H., Hirai T., Kim Y.H. (2019). Clinical impact of low serum free T4 in patients with non-small cell lung cancer treated with nivolumab. Sci. Rep..

[116] Sbardella E., Tenuta M., Sirgiovanni G., Gianfrilli D., Pozza C., Venneri M.A., Cortesi E., Marchetti P., Lenzi A., Gelibter A.J. (2020). Thyroid disorders in programmed death 1 inhibitor-treated patients: Is previous therapy with tyrosine kinase inhibitors a predisposing factor?. Clin. Endocrinol..

[117] Ahmadieh H., Salti I. (2013). Tyrosine Kinase Inhibitors Induced Thyroid Dysfunction: A Review of Its Incidence, Pathophysiology, Clinical Relevance, and Treatment. BioMed Res. Int..

[118] Funakoshi T., Shimada Y.J. (2013). Risk of hypothyroidism in patients with cancer treated with sunitinib: A systematic review and meta-analysis. Acta Oncol..

[119] Makita N., Iiri T. (2013). Tyrosine Kinase Inhibitor–Induced Thyroid Disorders: A Review and Hypothesis. Thyroid.

[120] Soni S., Rastogi A., Prasad K.T., Behera D., Singh N. (2021). Thyroid dysfunction in non-small cell lung cancer patients treated with epidermal growth factor receptor and anaplastic lymphoma kinase inhibitors: Results of a prospective cohort. Lung Cancer.

[121] Bilen M.A., Patel A., Hess K.R., Munoz J., Busaidy N.L., Wheler J.J., Janku F., Falchook G.S., Hong D.S., Meric-Bernstam F. (2016). Association between new-onset hypothyroidism and clinical response in patients treated with tyrosine kinase inhibitor therapy in phase I clinical trials. Cancer Chemother. Pharmacol..

[122] Si X., Zhang L., Wang H., Zhang X., Wang M., Han B., Li K., Wang Q., Shi J., Wang Z. (2019). Management of anlotinib-related adverse events in patients with advanced non-small cell lung cancer: Experiences in ALTER-0303. Thorac. Cancer.

[123] DeGroot L.J., Feingold K.R., Anawalt B. (2000). The Non-Thyroidal Illness Syndrome. Endotext.

[124] Schwarz Y., Percik R., Oberman B., Yaffe D., Zimlichman E., Tirosh A. (2021). Sick Euthyroid Syndrome on Presentation of Patients with COVID-19: A Potential Marker for Disease Severity. Endocr. Pract..

[125] Ratcliffe J.G., Stack B.H.R., Burt R.W., Ratcliffe W.A., Spilg W.G.S., Cuthbert J., Kennedy R.S. (1978). Thyroid function in lung cancer. Br. Med. J..

[126] EkremCengiz S., Cetinkaya E., Altin S., Gunluoglu Z., Demir A., Gunluoglu G., Epozturk K. (2008). Nutritional and Prognostic Significance of Sick Euthyroid Syndrome in Non-small Cell Lung Cancer Patients. Intern. Med..

[127] Yasar Z.A., Kirakli C., Yilmaz U., Ucar Z.Z., Talay F. (2014). Can Non-Thyroid Illness Syndrome Predict Mortality in Lung Cancer Patients? A Prospective Cohort Study. Horm. Cancer.

[128] Al-Farsi A., Ellis P.M. (2014). Treatment Paradigms for Patients with Metastatic Non-Small Cell Lung Cancer, Squamous Lung Cancer: First, Second, and Third-Line. Front. Oncol..

[129] Hijazo-Pechero S., Alay A., Marín R., Vilariño N., Muñoz-Pinedo C., Villanueva A., Santamaría D., Nadal E., Solé X. (2021). Gene Expression Profiling as a Potential Tool for Precision Oncology in Non-Small Cell Lung Cancer. Cancers.

[130] Nixon A.B., Schalper K.A., Jacobs I., Potluri S., Wang I.-M., Fleener C. (2019). Peripheral immune-based biomarkers in cancer immunotherapy: Can we realize their predictive potential?. J. Immunother. Cancer.

[131] Schiera G., di Liegro C.M., di Liegro I. (2021). Involvement of Thyroid Hormones in Brain Development and Cancer. Cancers.

[132] Piras C., Pibiri M., Leoni V.P., Balsamo A., Tronci L., Arisci N., Mariotti S., Atzori L. (2021). Analysis of metabolomics profile in hypothyroid patients before and after thyroid hormone replacement. J. Endocrinol. Investig..

[133] https://clinicaltrials.gov/ct2/show/NCT03823859.

[134] Rodríguez-Molinero A., Hercbergs A., Sarrias M., Yuste A. (2018). Plasma 3,3′,5-Triiodo-L-thyronine [T3] level mirrors changes in tumor markers in two cases of metastatic cancer of the breast and pancreas treated with exogenous L-T3. Cancer Biomark..

[135] Zhou X., Jiao L., Qian Y., Dong Q., Sun Y., Zheng W.V., Zhao W., Zhai W., Qiu L., Wu Y. (2021). Repositioning Azelnidipine as a Dual Inhibitor Targeting CD47/SIRPα and TIGIT/PVR Pathways for Cancer Immuno- Therapy. Biomolecules.

[136] Stevens D., Ingels J., Van Lint S., Vandekerckhove B., Vermaelen K. (2021). Dendritic Cell-Based Immunotherapy in Lung Cancer. Front. Immunol..

[137] Huang J., Ji G., Xing L., Li H., Wang Z., Ren G., Wu K., Kong L. (2013). Neo-endocrinochemotherapy: A novel approach for enhancing chemotherapeutic efficacy in clinic?. Med. Hypotheses.

[138] van Tassell B., Wohlford G.F., Linderman J.D., Smith S., Yavuz S., Pucino F., Celi F.S. (2019). Pharmacokinetics of L-Triiodothyronine in Patients Undergoing Thyroid Hormone Therapy Withdrawal. Thyroid.

[139] Columbano A., Chiellini G., Kowalik M.A. (2017). GC-1: A Thyromimetic with Multiple Therapeutic Applications in Liver Disease. Gene Expr..

[140] Köhrle J., Lehmphul I., Pietzner M., Renko K., Rijntjes E., Richards K., Anselmo J., Danielsen M., Jonklaas J. (2020). 3,5-T2—A Janus-Faced Thyroid Hormone Metabolite Exerts Both Canonical T3-Mimetic Endocrine and Intracrine Hepatic Action. Front. Endocrinol..

[141] Leith J.T., Mousa S.A., Hercbergs A., Lin H.Y., Davis P.J. (2018). Radioresistance of cancer cells, integrin αvβ3 and thyroid hormone. Oncotarget.

[142] Moossavi M., Parsamanesh N., Bahrami A., Atkin S.L., Sahebkar A. (2018). Role of the NLRP3 inflammasome in cancer. Mol. Cancer.

[143] Glinskii A.B., Glinsky G.V., Lin H.-Y., Tang H.-Y., Sun M., Davis F.B., Luidens M.K., Mousa S.A., Hercbergs A.H., Davis P.J. (2009). Modification of survival pathway gene expression in human breast cancer cells by tetraiodothyroacetic acid (tetrac). Cell Cycle.

[144] Ahmadi R., Ebrahimzadeh M.A. (2020). Resveratrol–A comprehensive review of recent advances in anticancer drug design and development. Eur. J. Med. Chem..

[145] Gorvel L., Olive D. (2020). Targeting the “PVR-TIGIT axis” with immune checkpoint therapies. F1000Research.

[146] Ghimessy A., Radeczky P., Laszlo V., Hegedus B., Renyi-Vamos F., Fillinger J., Klepetko W., Lang C., Dome B., Megyesfalvi Z. (2020). Current therapy of KRAS-mutant lung cancer. Cancer Metastasis Rev..

[147] Yang H., Liang S.-Q., Schmid R.A., Peng R.-W. (2019). New Horizons in KRAS-Mutant Lung Cancer: Dawn after Darkness. Front. Oncol..

[148] Ruggeri R.M., Campennì A., Deandreis D., Siracusa M., Tozzoli R., Ovčariček P.P., Giovanella L. (2021). SARS-CoV-2-related immune-inflammatory thyroid disorders: Facts and perspectives. Expert. Rev. Clin. Immunol..

[149] Speer G., Somogyi P. (2021). Thyroid complications of SARS and coronavirus disease 2019 (COVID-19). Endocr. J..

[150] Lui D.T.W., Lee C.H., Chow W.S., Lee A.C.H., Tam A.R., Fong C.H.Y., Law C.Y., Leung E.K.H., To K.K.W., Tan K.C.B. (2021). Thyroid Dysfunction in Relation to Immune Profile, Disease Status, and Outcome in 191 Patients with COVID-19. J. Clin. Endocrinol. Metab..

[151] Addeo A., Friedlaender A. (2020). Cancer and COVID-19: Unmasking their ties. Cancer Treat. Rev..

[152] Kuderer N.M., Choueiri T.K., Shah D.P., Shyr Y., Rubinstein S.M., Rivera D.R., Shete S., Hsu C.-Y., Desai A., de Lima Lopes G. (2020). COVID-19 and Cancer Consortium. Clinical impact of COVID-19 on patients with cancer (CCC19): A cohort study. Lancet.

[153] Tagliamento M., Agostinetto E., Bruzzone M., Ceppi M., Saini K.S., de Azambuja E., Punie K., Westphalen C.B., Morgan G., Pronzato P. (2021). Mortality in adult patients with solid or hematological malignancies and SARS-CoV-2 infection with a specific focus on lung and breast cancers: A systematic review and meta-analysis. Crit. Rev. Oncol. Hematol..

[154] Luo J., Rizvi H., Preeshagul I.R., Egger J.V., Hoyos D., Bandlamudi C., McCarthy C.G., Falcon C.J., Schoenfeld A.J., Arbour K.C. (2020). COVID-19 in patients with lung cancer. Ann. Oncol..

[155] Benderra M.-A., Aparicio A., Leblanc J., Wassermann D., Kempf E., Galula G., Bernaux M., Canellas A., Moreau T., Bellamine A. (2021). Clinical Characteristics, Care Trajectories and Mortality Rate of SARS-CoV-2 Infected Cancer Patients: A Multicenter Cohort Study. Cancers.

[156] Tao S.-L., Wang X.-M., Feng Y.-G., Kang P.-M., Li Q.-Y., Sun T.-Y., Tan Q.-Y., Deng B. (2020). Is the presence of lung injury in COVID-19 an independent risk factor for secondary lung cancer?. Med. Hypotheses.

[157] Davis P.J., Lin H.Y., Hercbergs A., Keating K.A., Mousa S.A. (2020). Coronaviruses and Integrin αvβ3: Does Thyroid Hormone Modify the Relationship?. Endocr. Res..

[158] Deligiorgi M.V., Siasos G., Vakkas L., Trafalis D.T. (2021). Charting the Unknown Association of COVID-19 with Thyroid Cancer, Focusing on Differentiated Thyroid Cancer: A Call for Caution. Cancers.

[159] Derosa L., Melenotte C., Griscelli F., Gachot B., Marabelle A., Kroemer G., Zitvogel L. (2020). The immuno-oncological challenge of COVID-19. Nat. Rev. Cancer.

[160] https://www.ncbi.nlm.nih.gov/research/coronavirus.

